# Supplementary material for: A Study on the Chemistry and Biological Activity of 26-Sulfur Analogs of Diosgenin: Synthesis of 26-Thiodiosgenin S-Mono- and Dioxides, and Their Alkyl Derivatives
Source: Molecules. 2022 Dec 26;28(1):189. doi: 10.3390/molecules28010189 (PMC9822051; doi:10.3390/molecules28010189)
Supplement: Supplementary file 1 [file molecules-28-00189-s001.zip › molecules-2099045-supplementary.pdf]

A study on the chemistry and biological activity of 26-sulfur analogs of diosgenin. Synthesis of 26-thiodiosgenin *S*-mono- and dioxides, and their alkyl derivatives

Aneta M. Tomkiel [1,*], Dorota Czajkowska-Szczykowska [1], Ewa Olchowik-Grabarek [2], Lucie Rárová [3], Szymon Sękowski [2] and Jacek W. Morzycki [1,*]

1 Laboratory of Natural Products, Department of Organic Chemistry, Faculty of Chemistry, University of Bialystok, K. Ciołkowskiego 1K, 15-245 Białystok, Poland
2 Laboratory of Molecular Biophysics, Department of Microbiology and Biotechnology, Faculty of Biology, University of Bialystok, K. Ciołkowskiego 1 J, 15-245 Białystok, Poland
3 Department of Experimental Biology, Faculty of Science, Palacký University, Šlechtitelů 27, CZ-78371 Olomouc, Czech Republic
* Correspondence: a.tomkiel@uwb.edu.pl (A.M.T.); morzycki@uwb.edu.pl (J.W.M.); Tel.: +48-85-738-80-44 (A.M.T.); +48-85-738-82-60 (J.W.M.)

Table of contents:

p. 3-4 Spectra of compound **3a**

p. 5-6 Spectra of compound **3b**

p. 7-8 Spectra of compound **6**

p. 9- 10 Spectra of compound **6a**

p. 11-12 Spectra of compound **6b**

p. 13-14 Spectra of compound **7**

p. 15-16 Spectra of compound **7a**

p. 17-18 Spectra of compound **8**

p. 19-20 Spectra of compound **8b**

p. 21-22 Spectra of compound **9**

p. 23-24 Spectra of compound **10**

p. 25-26 Spectra of compound **10b**

p. 27-28 Spectra of compound **11**

p. 29-30 Spectra of compound **11b**

p. 31-32 Spectra of compound **12**

p. 33-34 Spectra of compound **12b**

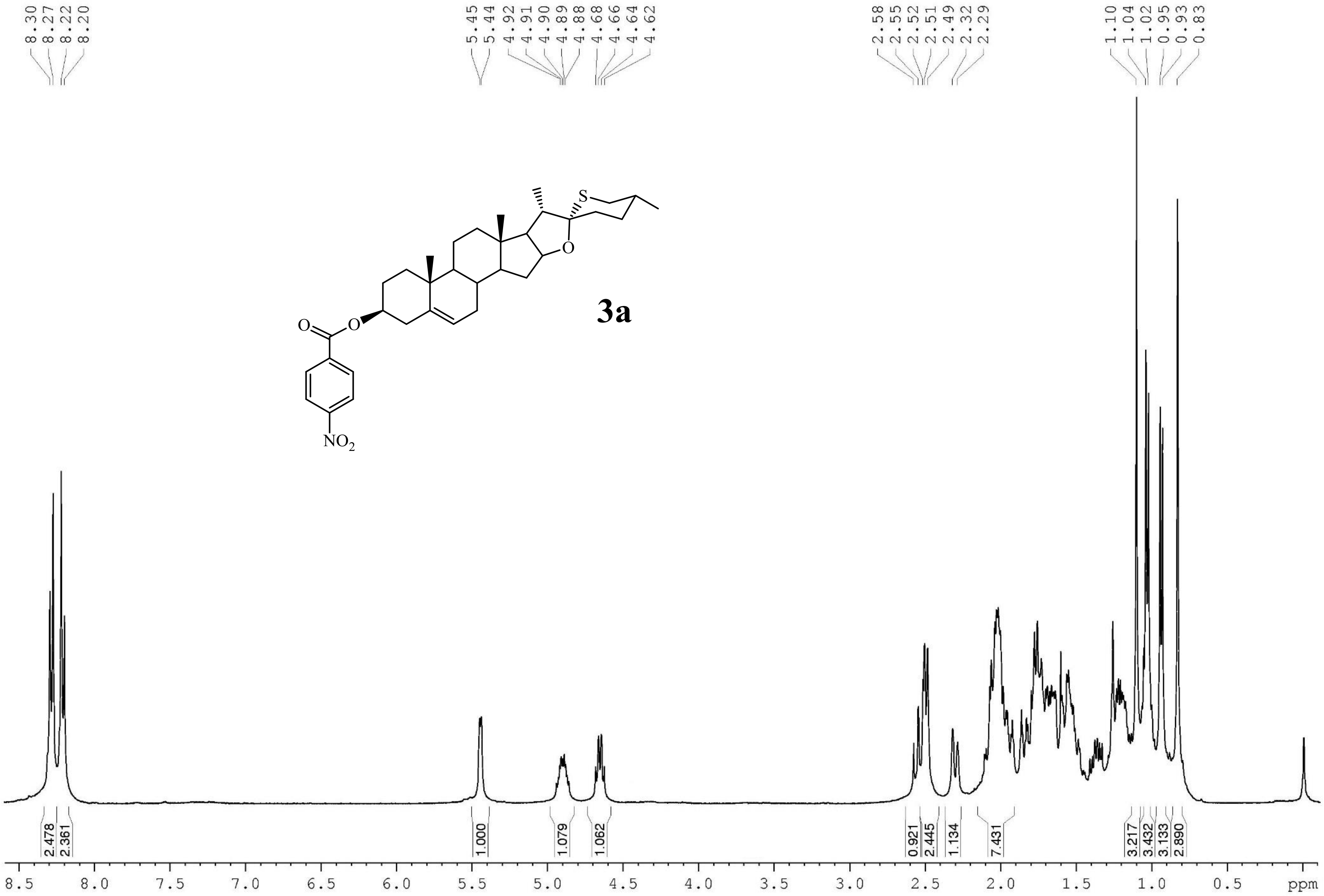
8.30 8.27 8.22 8.20
5.45 5.44 4.92 4.91 4.90 4.89 4.88 4.68 4.66 4.64 4.62
2.58 2.55 2.52 2.51 2.49 2.32 2.29
1.10 1.04 1.02 0.95 0.93 0.83
3a
2.478 2.361 1.000 1.079 1.062 0.921 2.445 1.134 7.431 3.217 3.432 3.133 2.890
8.5 8.0 7.5 7.0 6.5 6.0 5.5 5.0 4.5 4.0 3.5 3.0 2.5 2.0 1.5 1.0 0.5 ppm

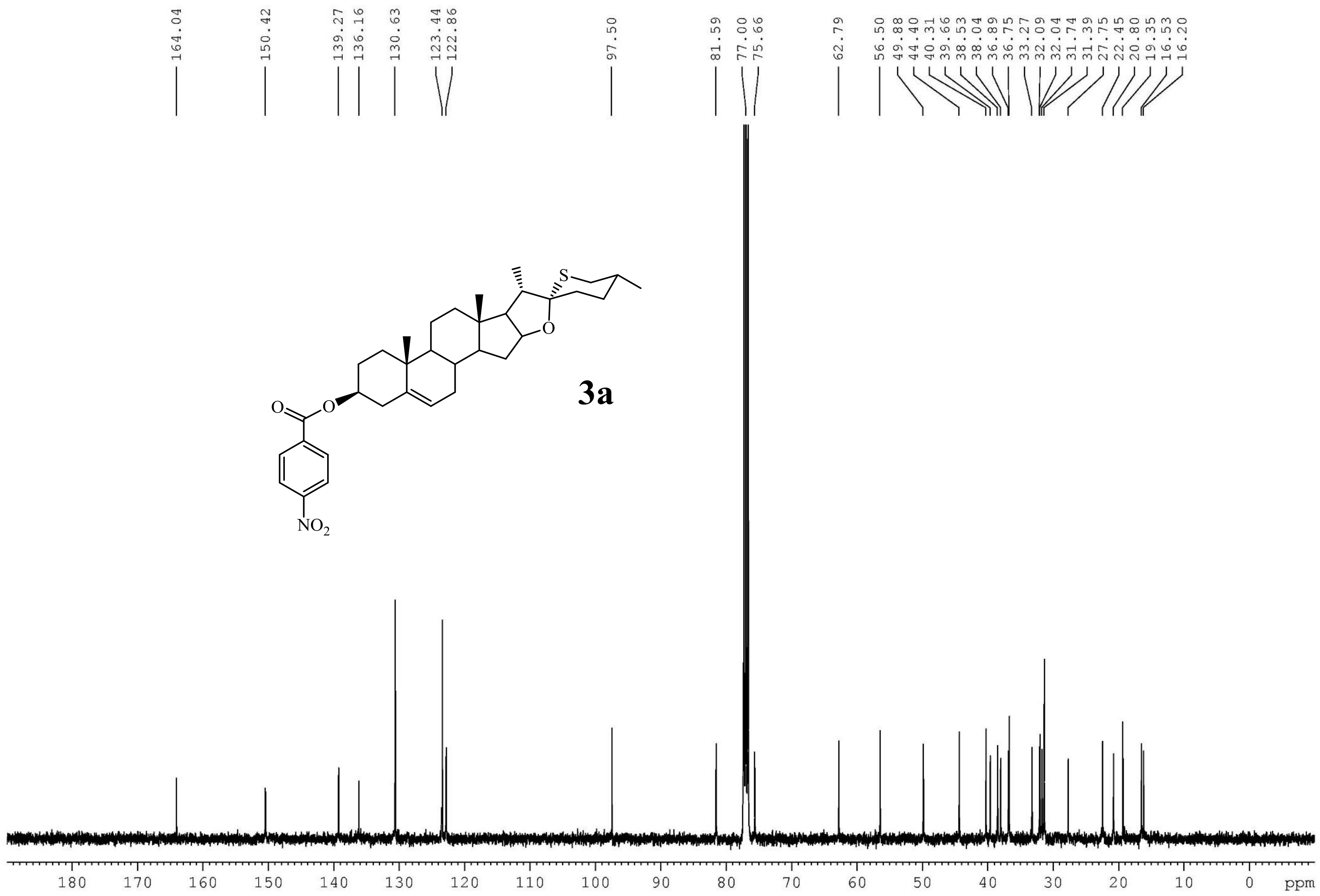
164.04
150.42
139.27
136.16
130.63
123.44
122.86
97.50
81.59
77.00
75.66
62.79
56.50
49.88
44.40
40.31
39.66
38.53
38.04
36.89
36.75
33.27
32.09
32.04
31.74
31.39
27.75
22.45
20.80
19.35
16.53
16.20
S
O
O
O
NO2
3a
180 170 160 150 140 130 120 110 100 90 80 70 60 50 40 30 20 10 0 ppm

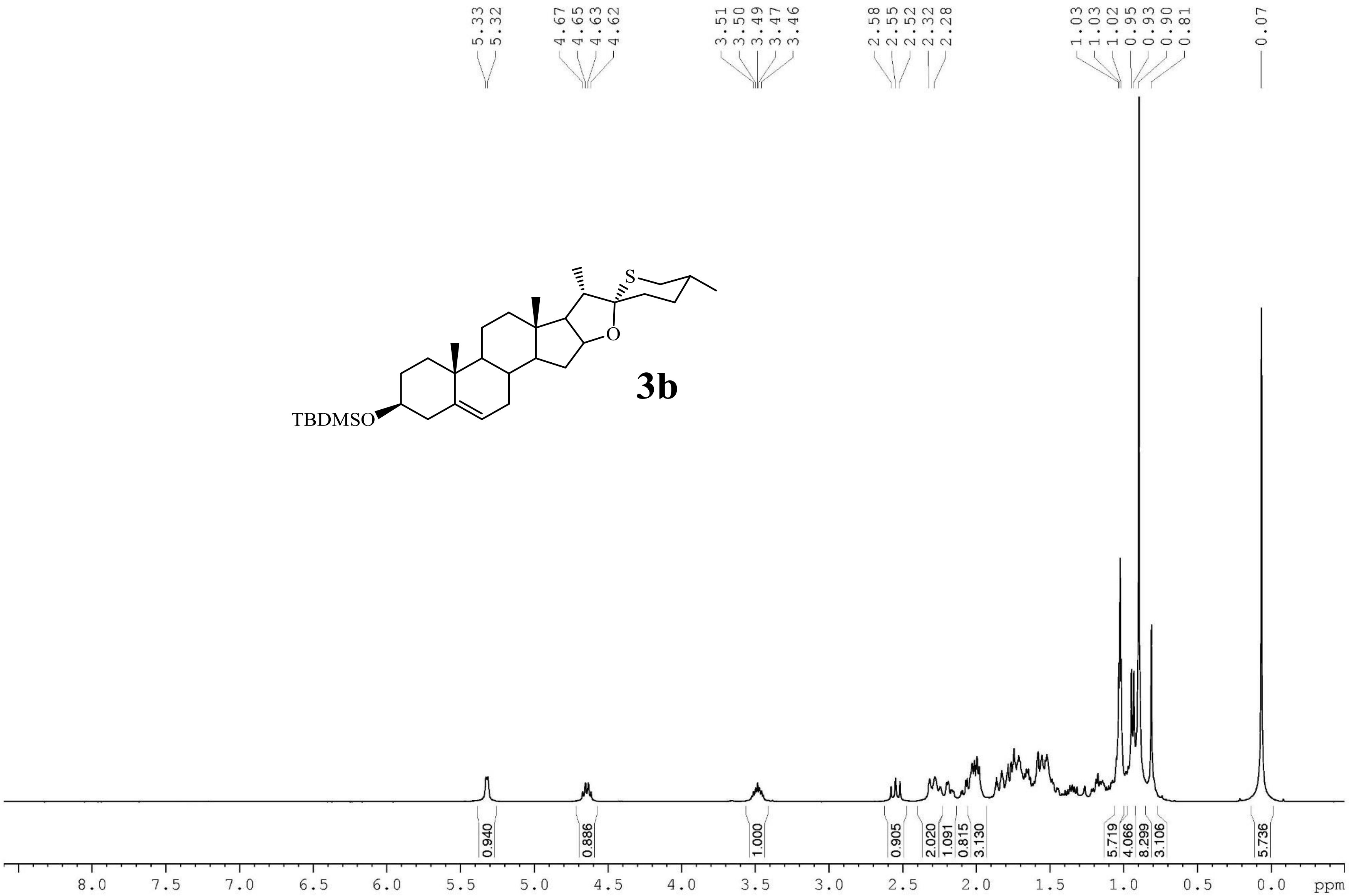
5.33
5.32
4.67
4.65
4.63
4.62
3.51
3.50
3.49
3.47
3.46
2.58
2.55
2.52
2.32
2.28
1.03
1.03
1.02
0.95
0.93
0.90
0.81
0.07
S
O
TBDMSO
3b
0.940
0.886
1.000
0.905
2.020
1.091
0.815
3.130
5.719
4.066
8.299
3.106
5.736
8.0
7.5
7.0
6.5
6.0
5.5
5.0
4.5
4.0
3.5
3.0
2.5
2.0
1.5
1.0
0.5
0.0
ppm

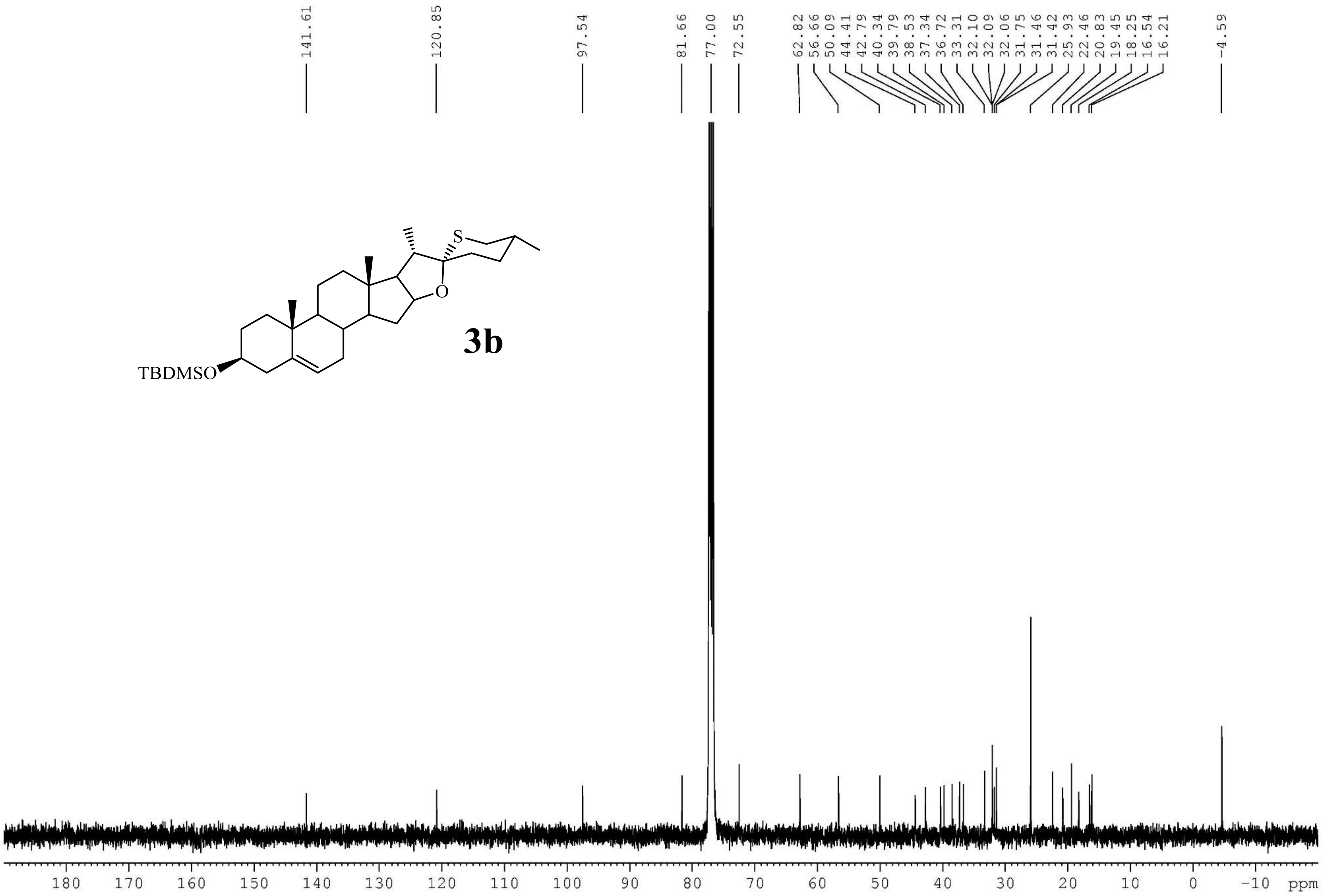
141.61
120.85
97.54
81.66
77.00
72.55
62.82
56.66
50.09
44.41
42.79
40.34
39.79
38.53
37.34
36.72
33.31
32.10
32.09
32.06
31.75
31.46
31.42
25.93
22.46
20.83
19.45
18.25
16.54
16.21
-4.59
TBDMSO
S
O
180 170 160 150 140 130 120 110 100 90 80 70 60 50 40 30 20 10 0 -10 ppm

3b

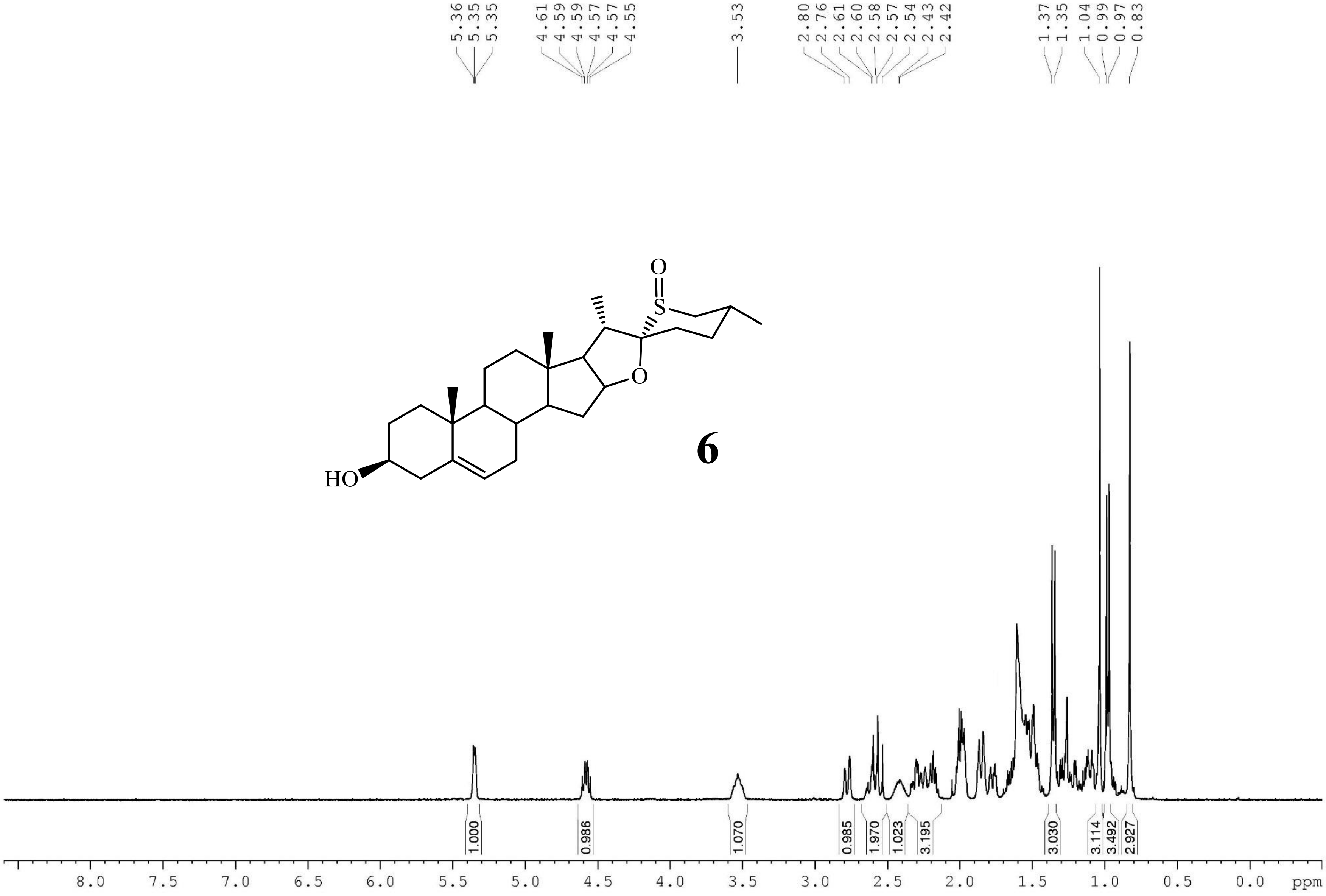
5.36
5.35
5.35
4.61
4.59
4.59
4.57
4.57
4.55
3.53
2.80
2.76
2.61
2.60
2.58
2.57
2.54
2.43
2.42
1.37
1.35
1.04
0.99
0.97
0.83
O
S
O
HO
6
1.000
0.986
1.070
0.985
1.970
1.023
3.195
3.030
3.114
3.492
2.927
8.0
7.5
7.0
6.5
6.0
5.5
5.0
4.5
4.0
3.5
3.0
2.5
2.0
1.5
1.0
0.5
0.0
ppm

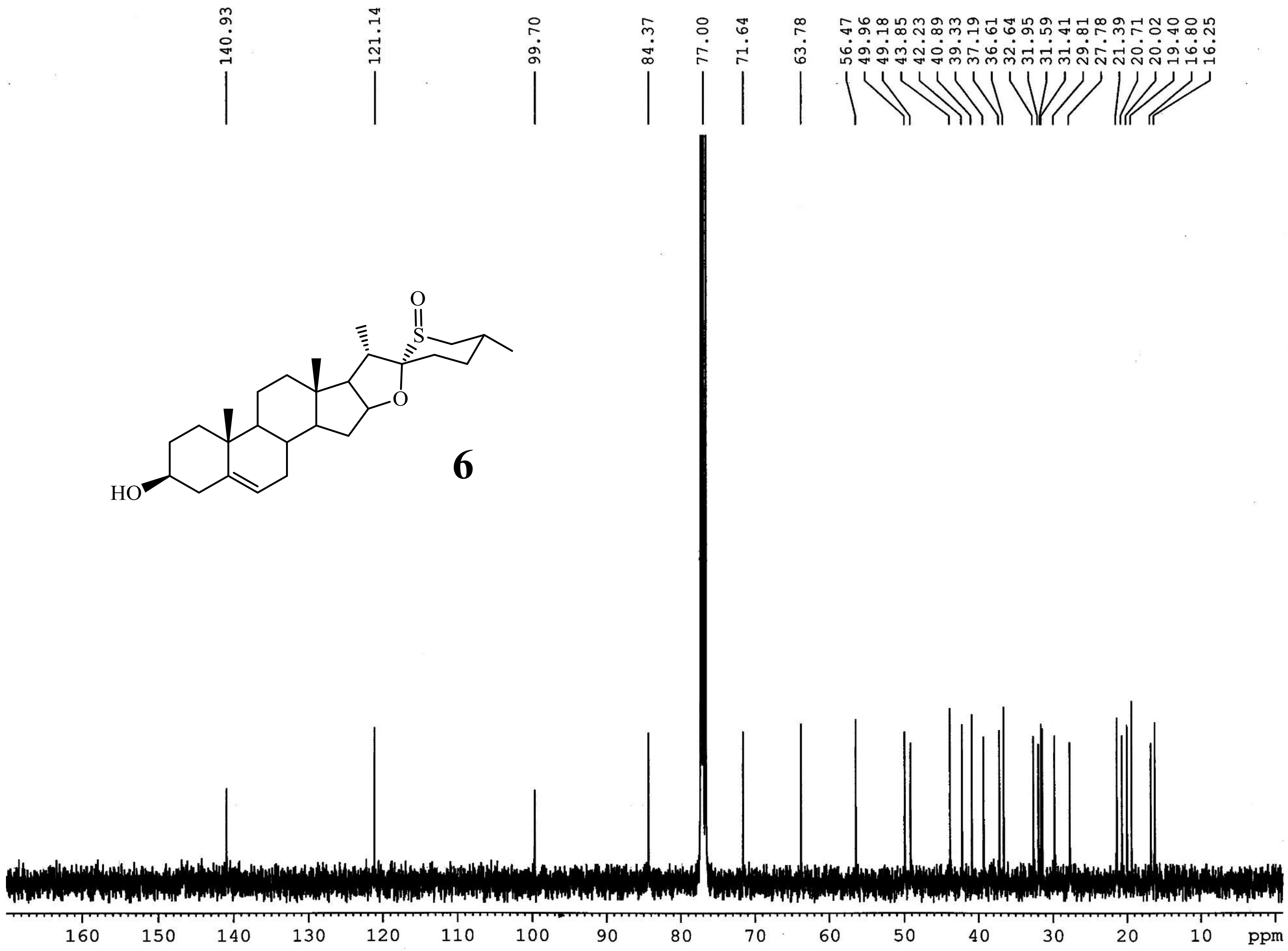
140.93
121.14
99.70
84.37
77.00
71.64
63.78
56.47
49.96
49.18
43.85
42.23
40.89
39.33
37.19
36.61
32.64
31.95
31.59
31.41
29.81
27.78
21.39
20.71
20.02
19.40
16.80
16.25
O
S
O
HO
6
160 150 140 130 120 110 100 90 80 70 60 50 40 30 20 10 ppm

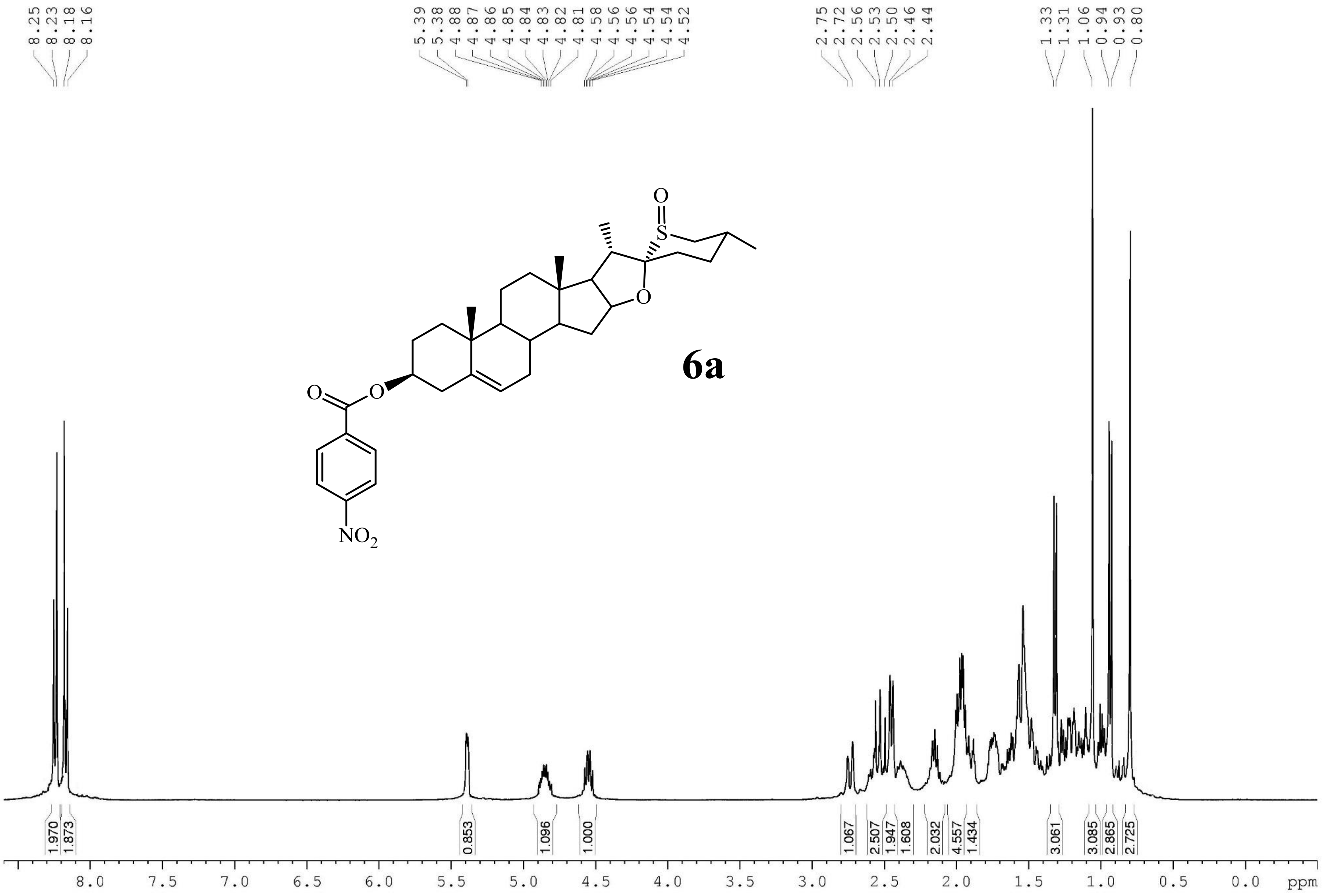
8.25 8.23 8.18 8.16
5.39 5.38 4.88 4.87 4.86 4.85 4.84 4.83 4.82 4.81 4.58 4.56 4.56 4.54 4.54 4.52
2.75 2.72 2.56 2.53 2.50 2.46 2.44
1.33 1.31 1.06 0.94 0.93 0.80
O
S
O
O
O
NO2
6a
1.970 1.873 0.853 1.096 1.000 1.067 2.507 1.947 1.608 2.032 4.557 1.434 3.061 3.085 2.865 2.725
8.0 7.5 7.0 6.5 6.0 5.5 5.0 4.5 4.0 3.5 3.0 2.5 2.0 1.5 1.0 0.5 0.0 ppm

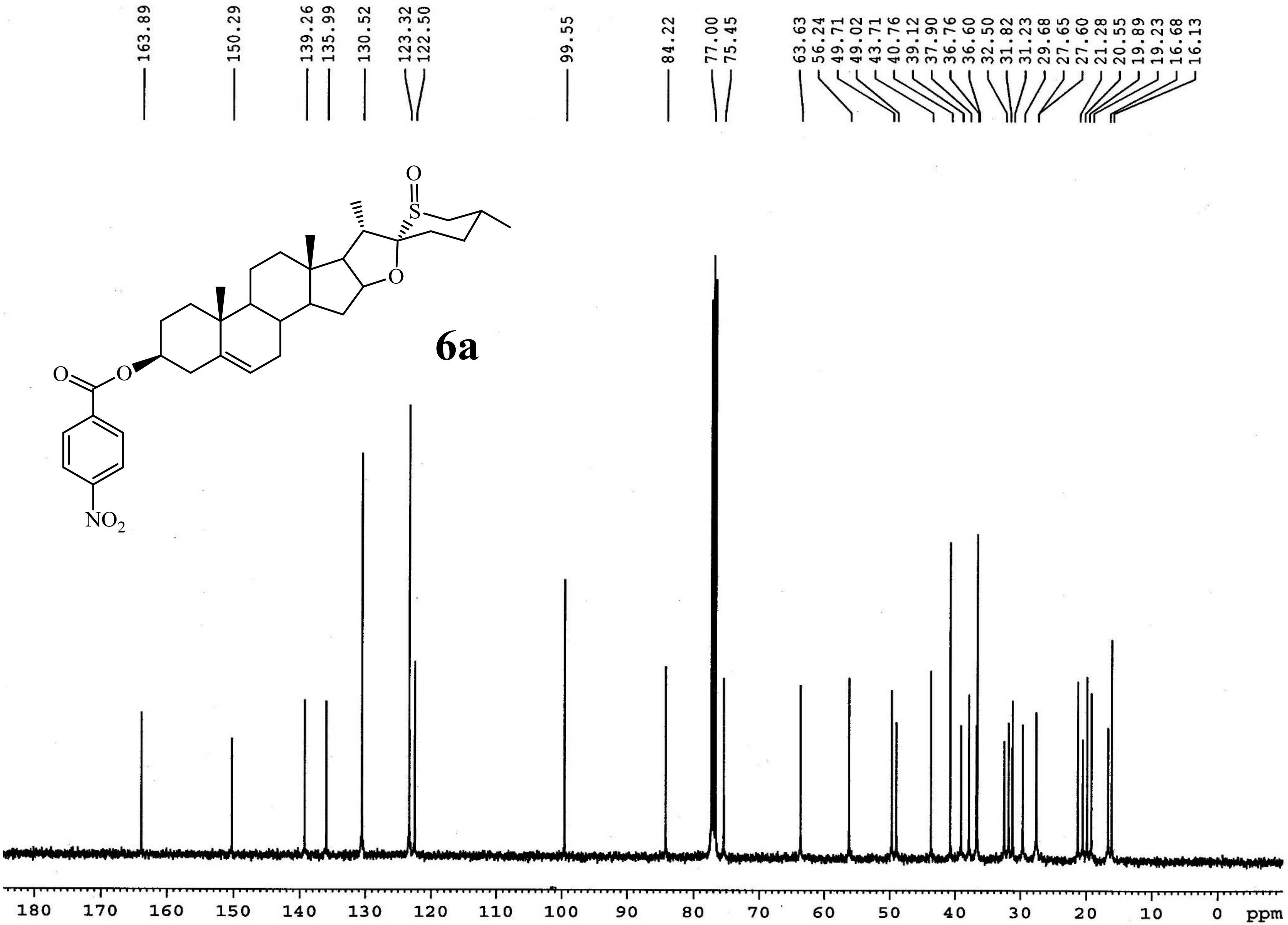
163.89
150.29
139.26
135.99
130.52
123.32
122.50
99.55
84.22
77.00
75.45
63.63
56.24
49.71
49.02
43.71
40.76
39.12
37.90
36.76
36.60
32.50
31.82
31.23
29.68
27.65
27.60
21.28
20.55
19.89
19.23
16.68
16.13
6a
NO_2
180 170 160 150 140 130 120 110 100 90 80 70 60 50 40 30 20 10 0 ppm

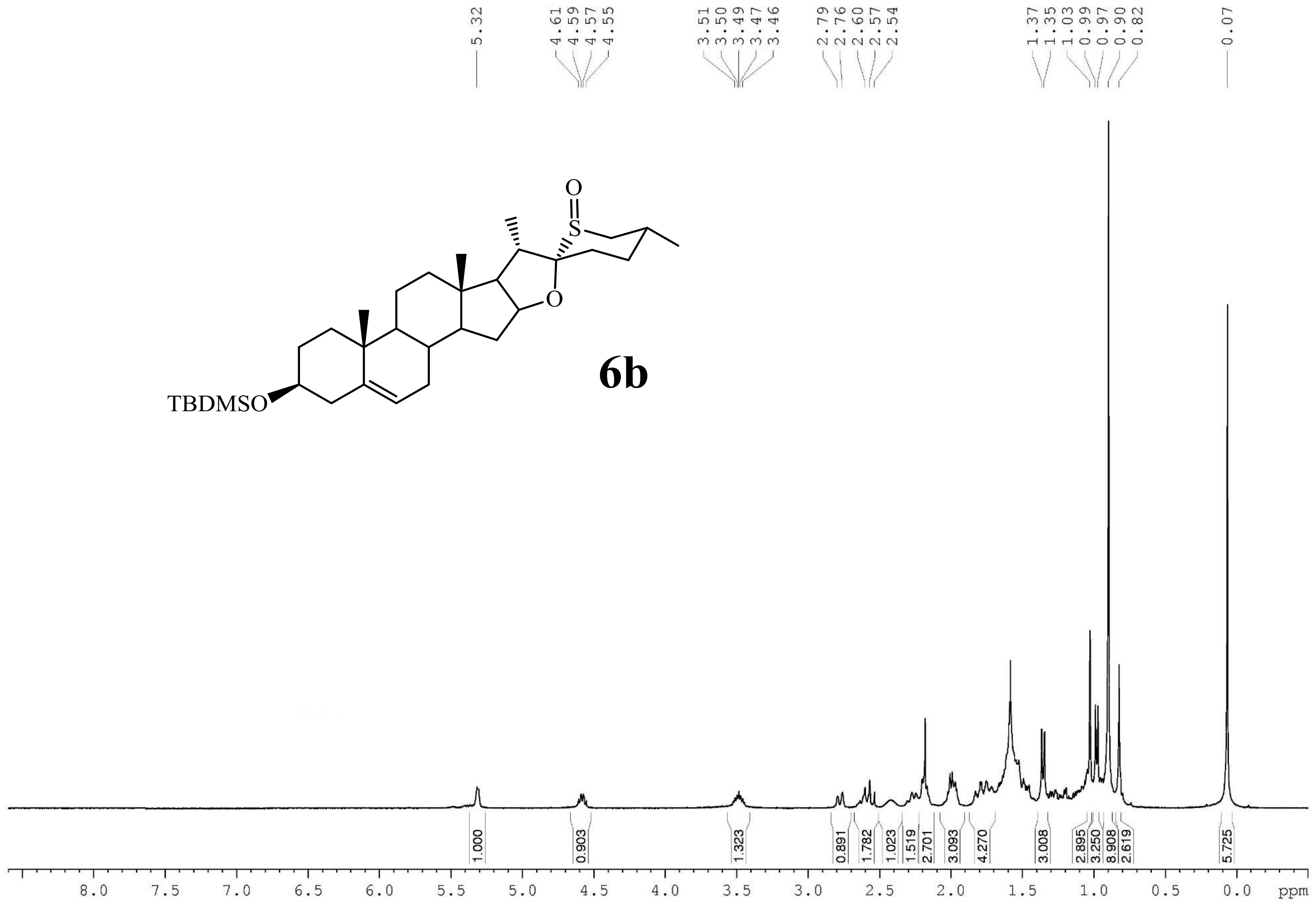
5.32
4.61 4.59 4.57 4.55
3.51 3.50 3.49 3.47 3.46
2.79 2.76 2.60 2.57 2.54
1.37 1.35 1.03 0.99 0.97 0.90 0.82
0.07
O
S
O
TBDMSO
6b
1.000
0.903
1.323
0.891 1.782 1.023 1.519 2.701 3.093 4.270
3.008
2.895 3.250 8.908 2.619
5.725
8.0 7.5 7.0 6.5 6.0 5.5 5.0 4.5 4.0 3.5 3.0 2.5 2.0 1.5 1.0 0.5 0.0 ppm

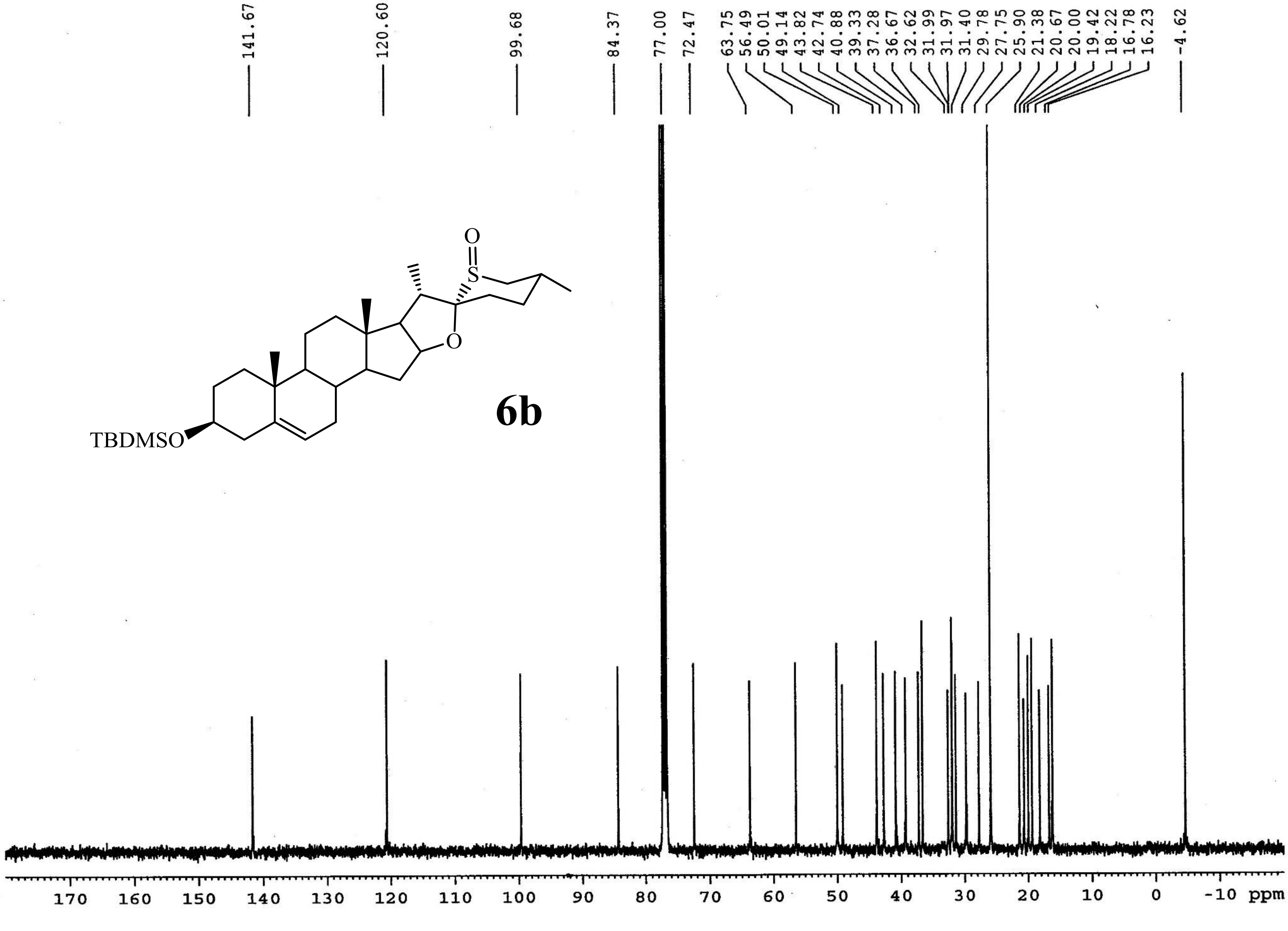
TBDMSO
6b
141.67
120.60
99.68
84.37
77.00
72.47
63.75
56.49
50.01
49.14
43.82
42.74
40.88
39.33
37.28
36.67
32.62
31.99
31.97
31.40
29.78
27.75
25.90
21.38
20.67
20.00
19.42
18.22
16.78
16.23
-4.62
170 160 150 140 130 120 110 100 90 80 70 60 50 40 30 20 10 0 -10 ppm

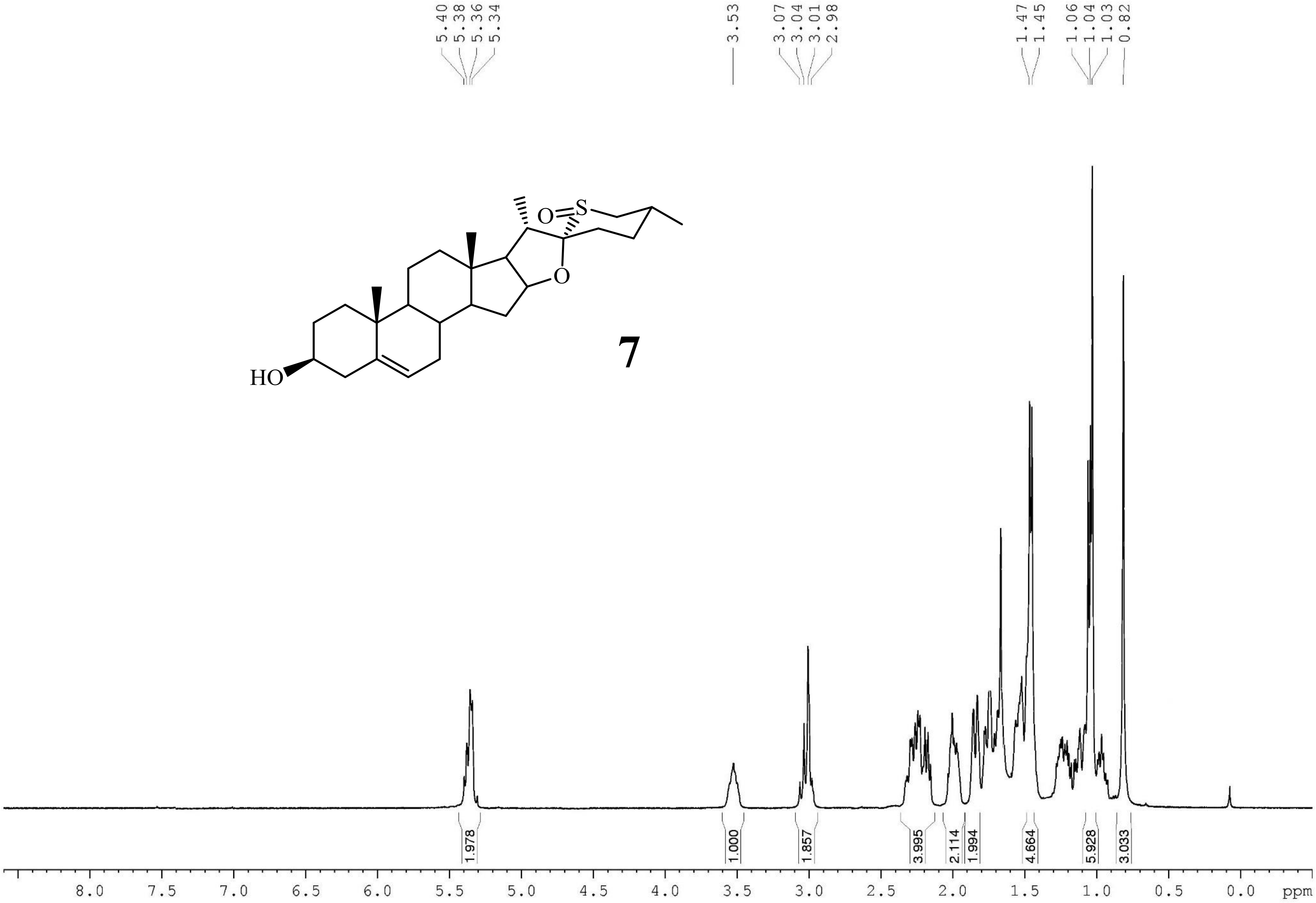
5.40
5.38
5.36
5.34
3.53
3.07
3.04
3.01
2.98
1.47
1.45
1.06
1.04
1.03
0.82
HO
O
O=S
7
1.978
1.000
1.857
3.995
2.114
1.994
4.664
5.928
3.033
8.0 7.5 7.0 6.5 6.0 5.5 5.0 4.5 4.0 3.5 3.0 2.5 2.0 1.5 1.0 0.5 0.0 ppm

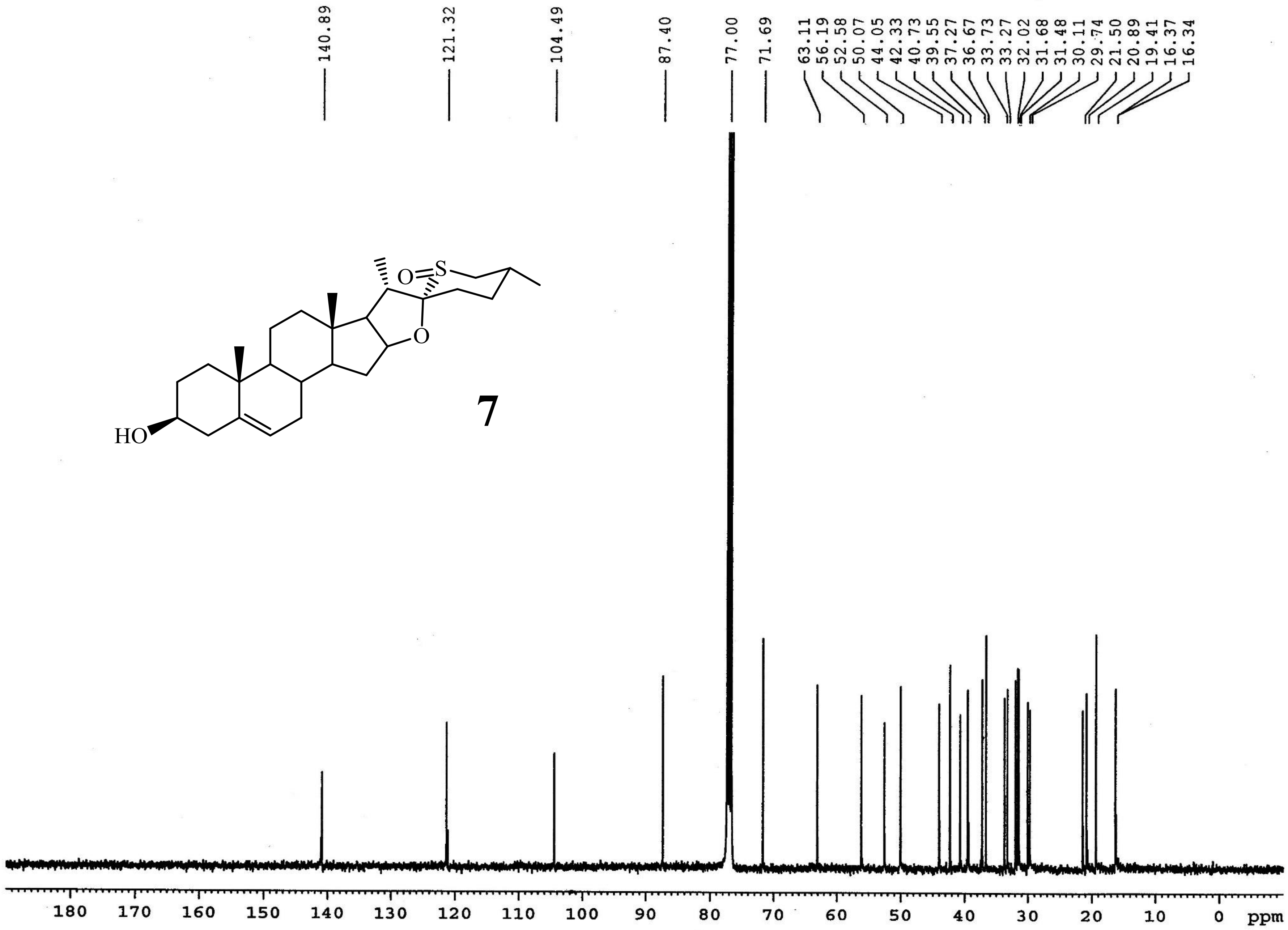
140.89
121.32
104.49
87.40
77.00
71.69
63.11
56.19
52.58
50.07
44.05
42.33
40.73
39.55
37.27
36.67
33.73
33.27
32.02
31.68
31.48
30.11
29.74
21.50
20.89
19.41
16.37
16.34
180 170 160 150 140 130 120 110 100 90 80 70 60 50 40 30 20 10 0 ppm

7

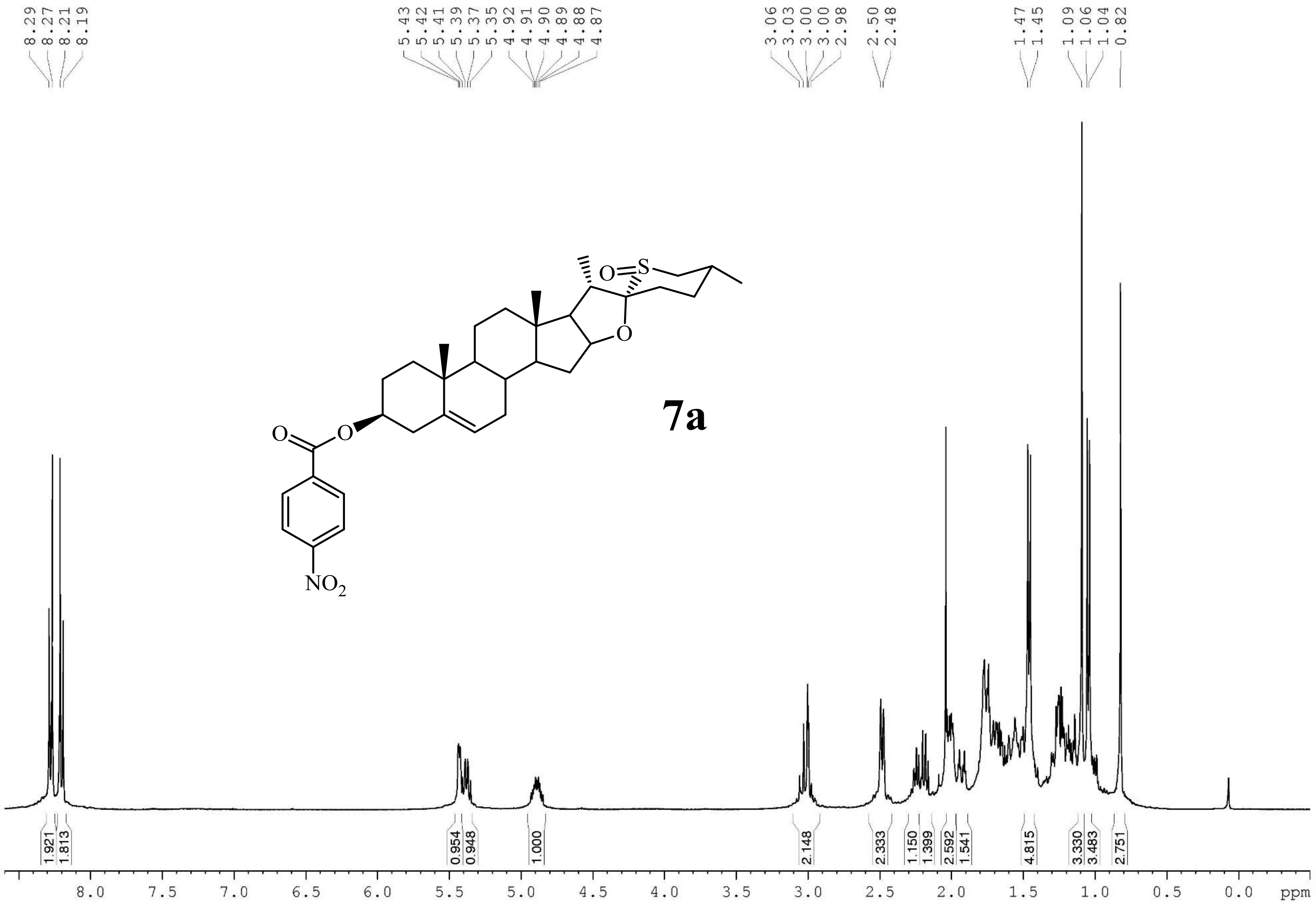
8.29
8.27
8.21
8.19
5.43
5.42
5.41
5.39
5.37
5.35
4.92
4.91
4.90
4.89
4.88
4.87
3.06
3.03
3.00
3.00
2.98
2.50
2.48
1.47
1.45
1.09
1.06
1.04
0.82
O=S
O
O
O
NO2
7a
1.921
1.813
0.954
0.948
1.000
2.148
2.333
1.150
1.399
2.592
1.541
4.815
3.330
3.483
2.751
8.0
7.5
7.0
6.5
6.0
5.5
5.0
4.5
4.0
3.5
3.0
2.5
2.0
1.5
1.0
0.5
0.0
ppm

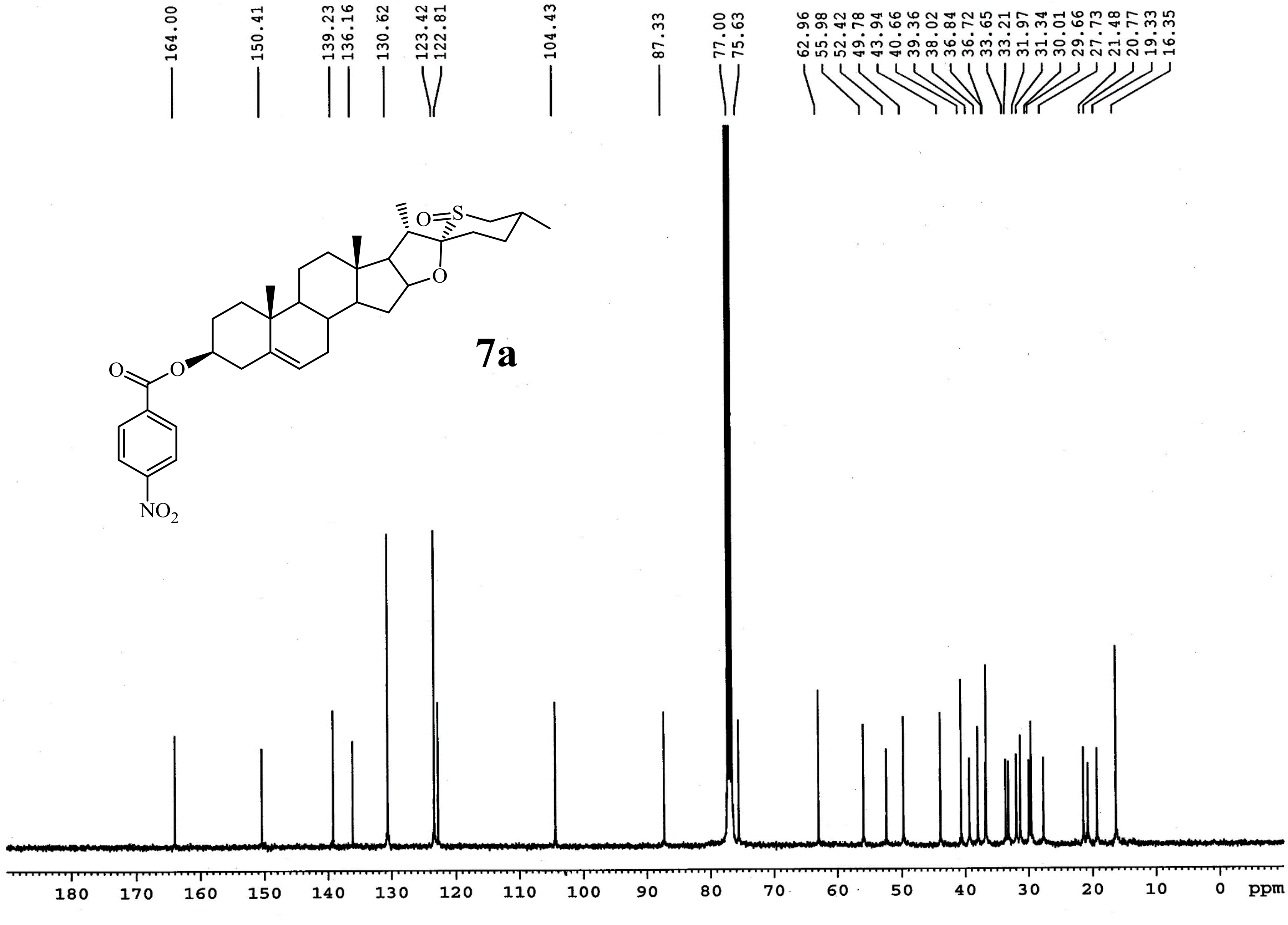
164.00
150.41
139.23
136.16
130.62
123.42
122.81
104.43
87.33
77.00
75.63
62.96
55.98
52.42
49.78
43.94
40.66
39.36
38.02
36.84
36.72
33.65
33.21
31.97
31.34
30.01
29.66
27.73
21.48
20.77
19.33
16.35
O
S
O
O
O
NO2
7a
180 170 160 150 140 130 120 110 100 90 80 70 60 50 40 30 20 10 0 ppm

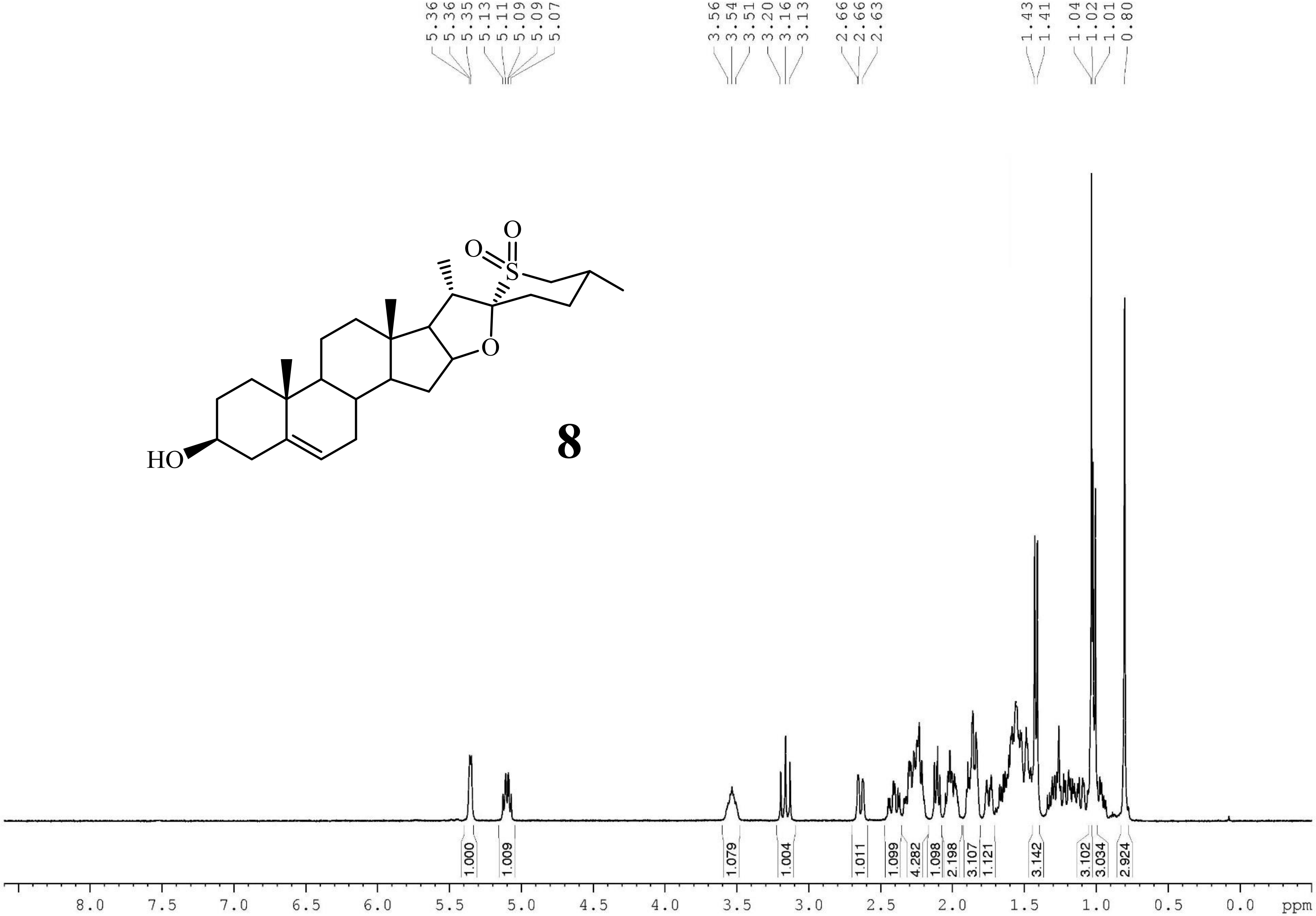
5.36
5.36
5.35
5.13
5.11
5.09
5.09
5.07
3.56
3.54
3.51
3.20
3.16
3.13
2.66
2.66
2.63
1.43
1.41
1.04
1.02
1.01
0.80
O
O
S
O
HO
1.000
1.009
1.079
1.004
1.011
1.099
4.282
1.098
2.198
3.107
1.121
3.142
3.102
3.034
2.924
8.0
7.5
7.0
6.5
6.0
5.5
5.0
4.5
4.0
3.5
3.0
2.5
2.0
1.5
1.0
0.5
0.0
ppm

8

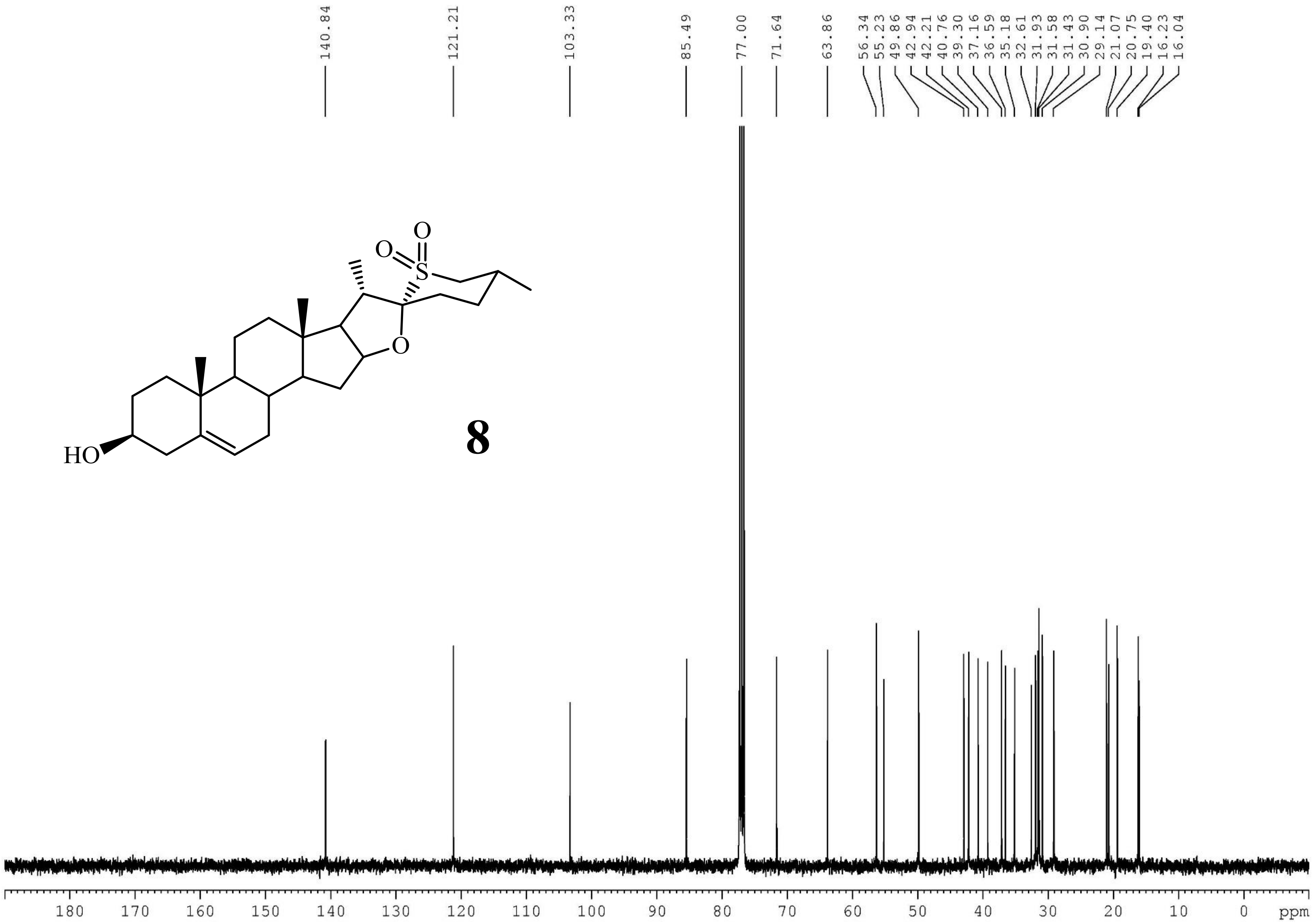
140.84
121.21
103.33
85.49
77.00
71.64
63.86
56.34
55.23
49.86
42.94
42.21
40.76
39.30
37.16
36.59
35.18
32.61
31.93
31.58
31.43
30.90
29.14
21.07
20.75
19.40
16.23
16.04
O
O
S
O
HO
180 170 160 150 140 130 120 110 100 90 80 70 60 50 40 30 20 10 0 ppm

8

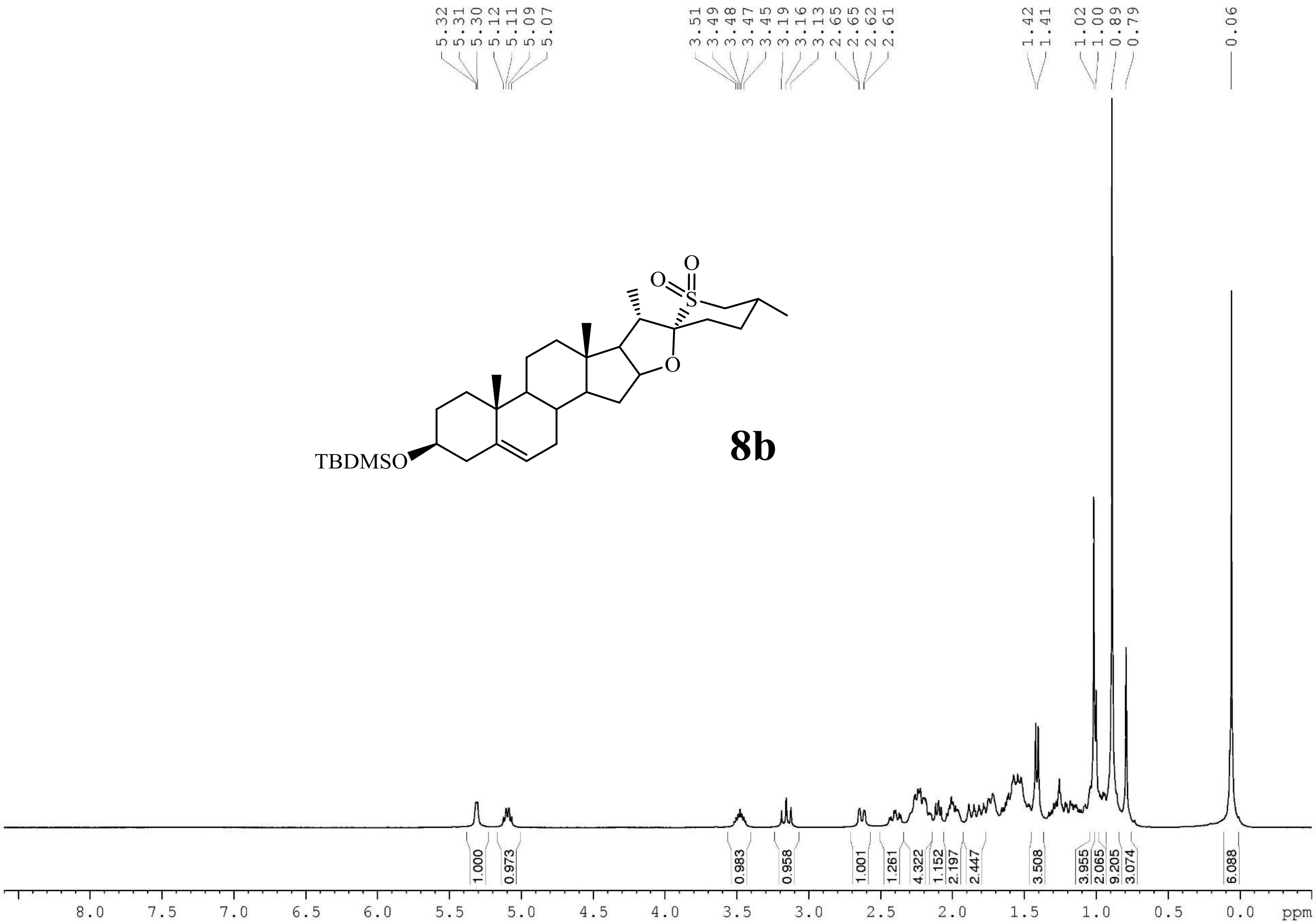
5.32 5.31 5.30 5.12 5.11 5.09 5.07
3.51 3.49 3.48 3.47 3.45 3.19 3.16 3.13 2.65 2.65 2.62 2.61
1.42 1.41
1.02 1.00 0.89 0.79
0.06
TBDMSO
O
S
8b
1.000 0.973 0.983 0.958 1.001 1.261 4.322 1.152 2.197 2.447 3.508 3.955 2.065 9.205 3.074 6.088
8.0 7.5 7.0 6.5 6.0 5.5 5.0 4.5 4.0 3.5 3.0 2.5 2.0 1.5 1.0 0.5 0.0 ppm

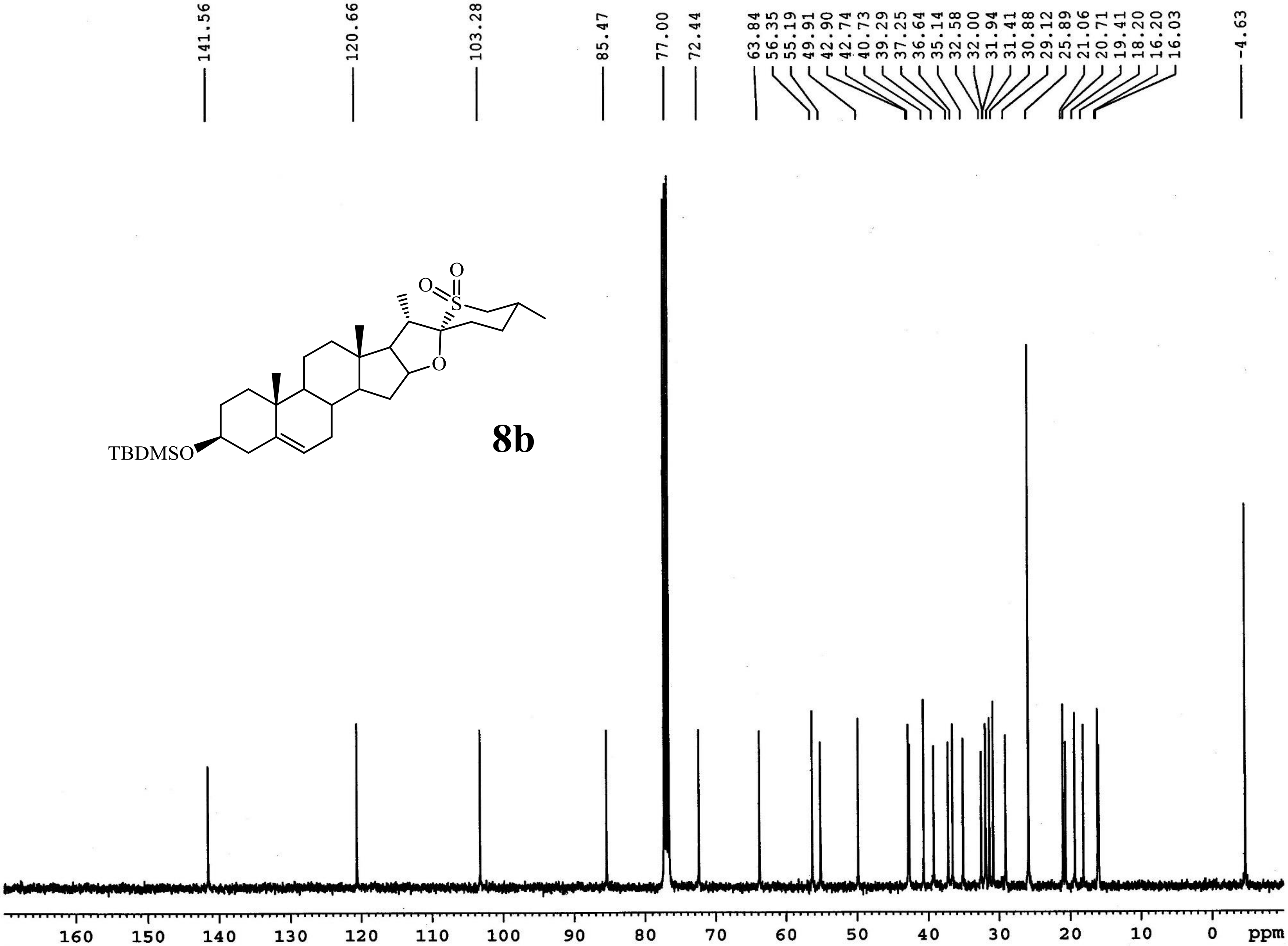
141.56
120.66
103.28
85.47
77.00
72.44
63.84
56.35
55.19
49.91
42.90
42.74
40.73
39.29
37.25
36.64
35.14
32.58
32.00
31.94
31.41
30.88
29.12
25.89
21.06
20.71
19.41
18.20
16.20
16.03
-4.63
TBDMSO
O
S
O
O
8b
160 150 140 130 120 110 100 90 80 70 60 50 40 30 20 10 0 ppm

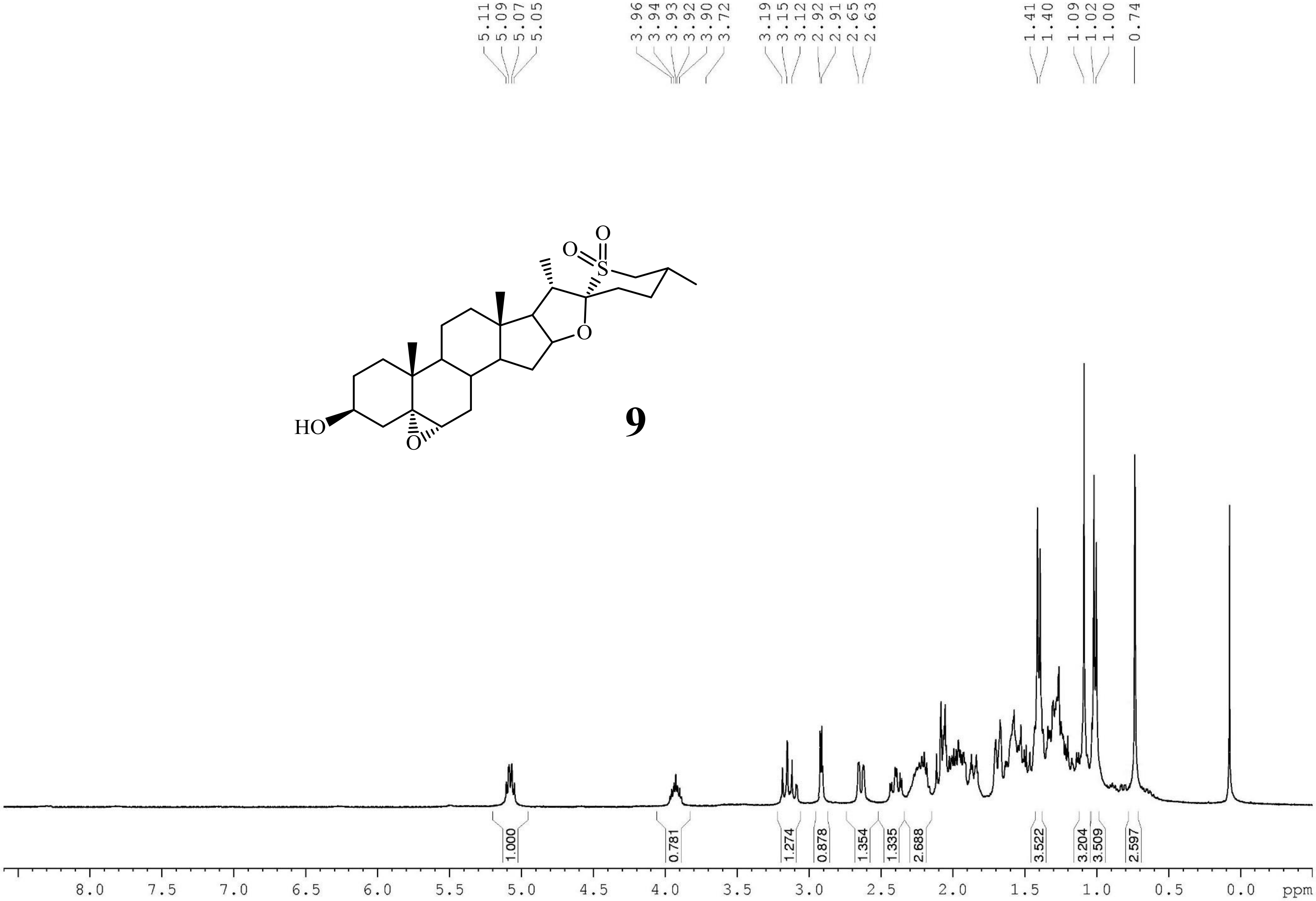
5.11
5.09
5.07
5.05
3.96
3.94
3.93
3.92
3.90
3.72
3.19
3.15
3.12
2.92
2.91
2.65
2.63
1.41
1.40
1.09
1.02
1.00
0.74
HO
O
O
O
S
O
1.000
0.781
1.274
0.878
1.354
1.335
2.688
3.522
3.204
3.509
2.597
8.0 7.5 7.0 6.5 6.0 5.5 5.0 4.5 4.0 3.5 3.0 2.5 2.0 1.5 1.0 0.5 0.0 ppm

9

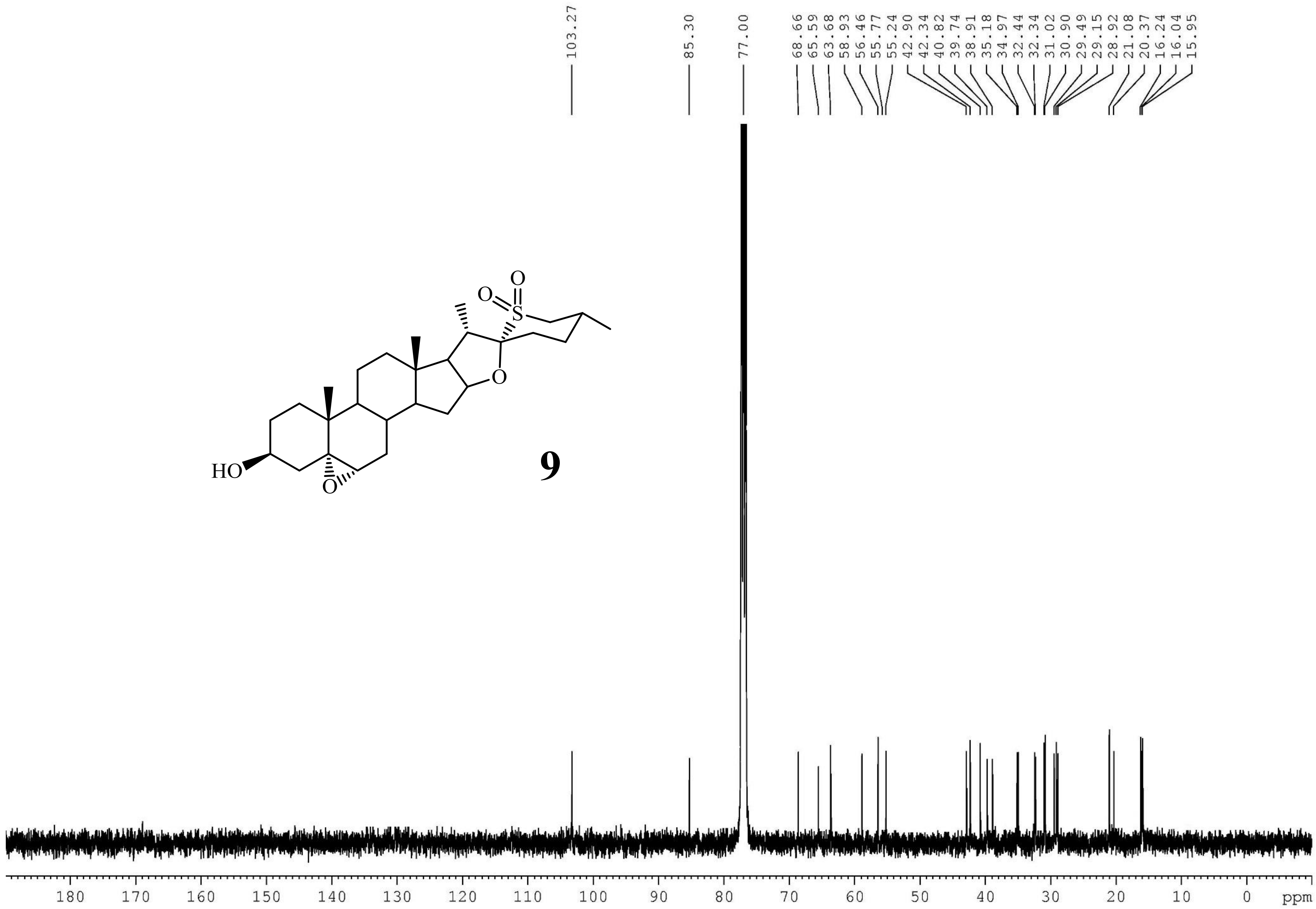

103.27
85.30
77.00
68.66
65.59
63.68
58.93
56.46
55.77
55.24
42.90
42.34
40.82
39.74
38.91
35.18
34.97
32.44
32.34
31.02
30.90
29.49
29.15
28.92
21.08
20.37
16.24
16.04
15.95
O
O
S
O
HO
O
9
180 170 160 150 140 130 120 110 100 90 80 70 60 50 40 30 20 10 0 ppm

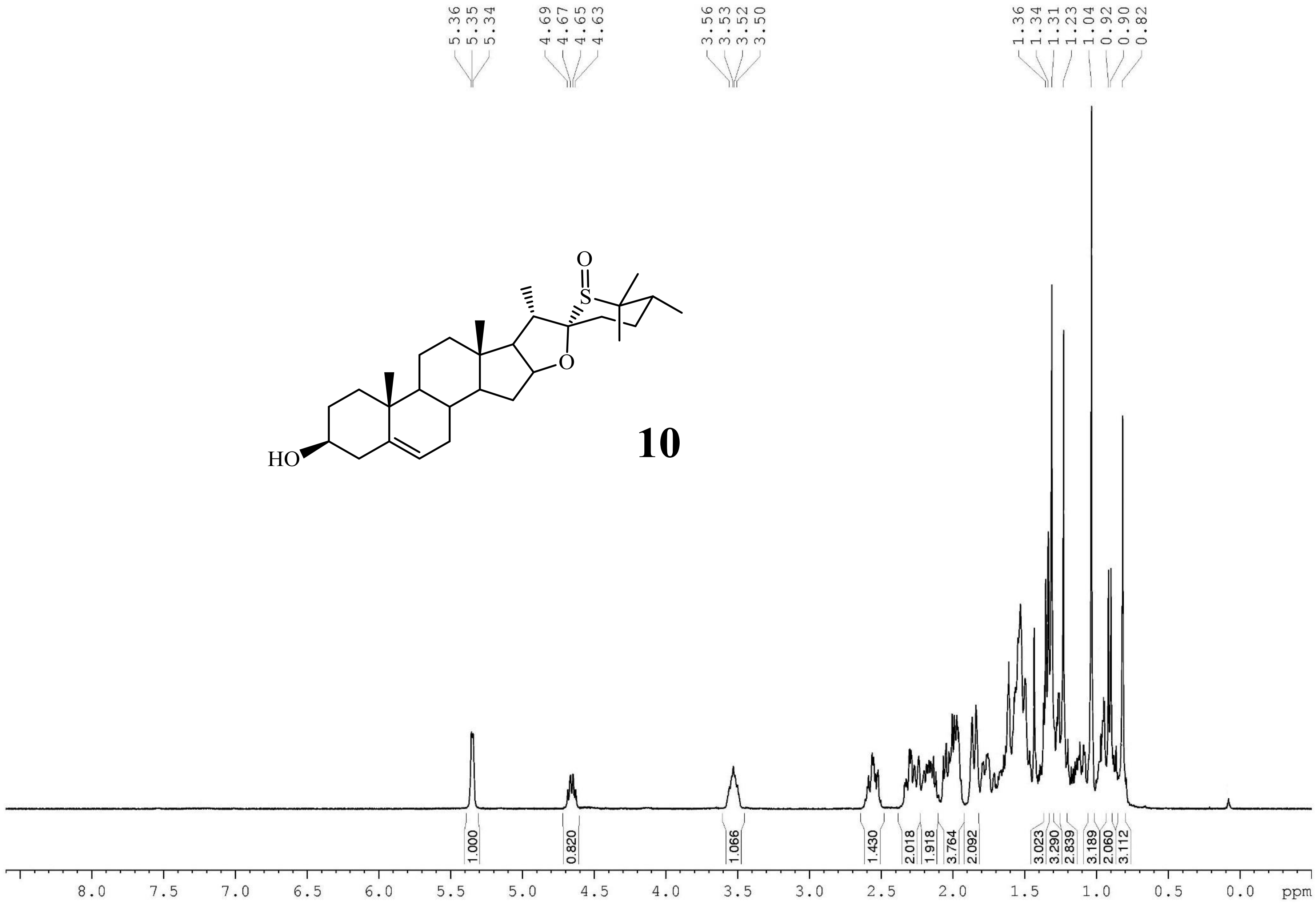
5.36
5.35
5.34
4.69
4.67
4.65
4.63
3.56
3.53
3.52
3.50
1.36
1.34
1.31
1.23
1.04
0.92
0.90
0.82
O
S
O
HO
10
1.000
0.820
1.066
1.430
2.018
1.918
3.764
2.092
3.023
3.290
2.839
3.189
2.060
3.112
8.0
7.5
7.0
6.5
6.0
5.5
5.0
4.5
4.0
3.5
3.0
2.5
2.0
1.5
1.0
0.5
0.0
ppm

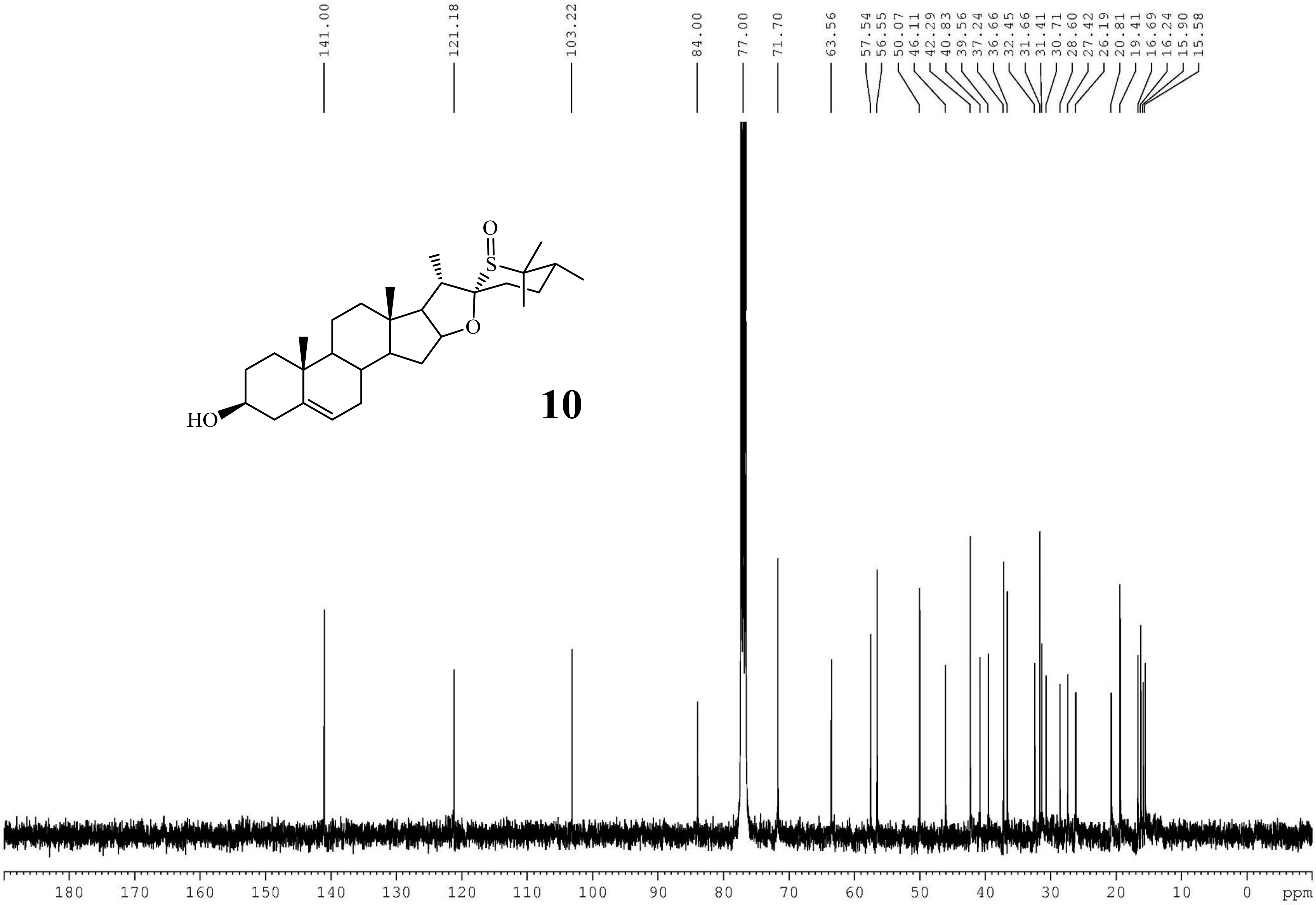
141.00
121.18
103.22
84.00
77.00
71.70
63.56
57.54
56.55
50.07
46.11
42.29
40.83
39.56
37.24
36.66
32.45
31.66
31.41
30.71
28.60
27.42
26.19
20.81
19.41
16.69
16.24
15.90
15.58
O
S
O
HO
180 170 160 150 140 130 120 110 100 90 80 70 60 50 40 30 20 10 0 ppm

10

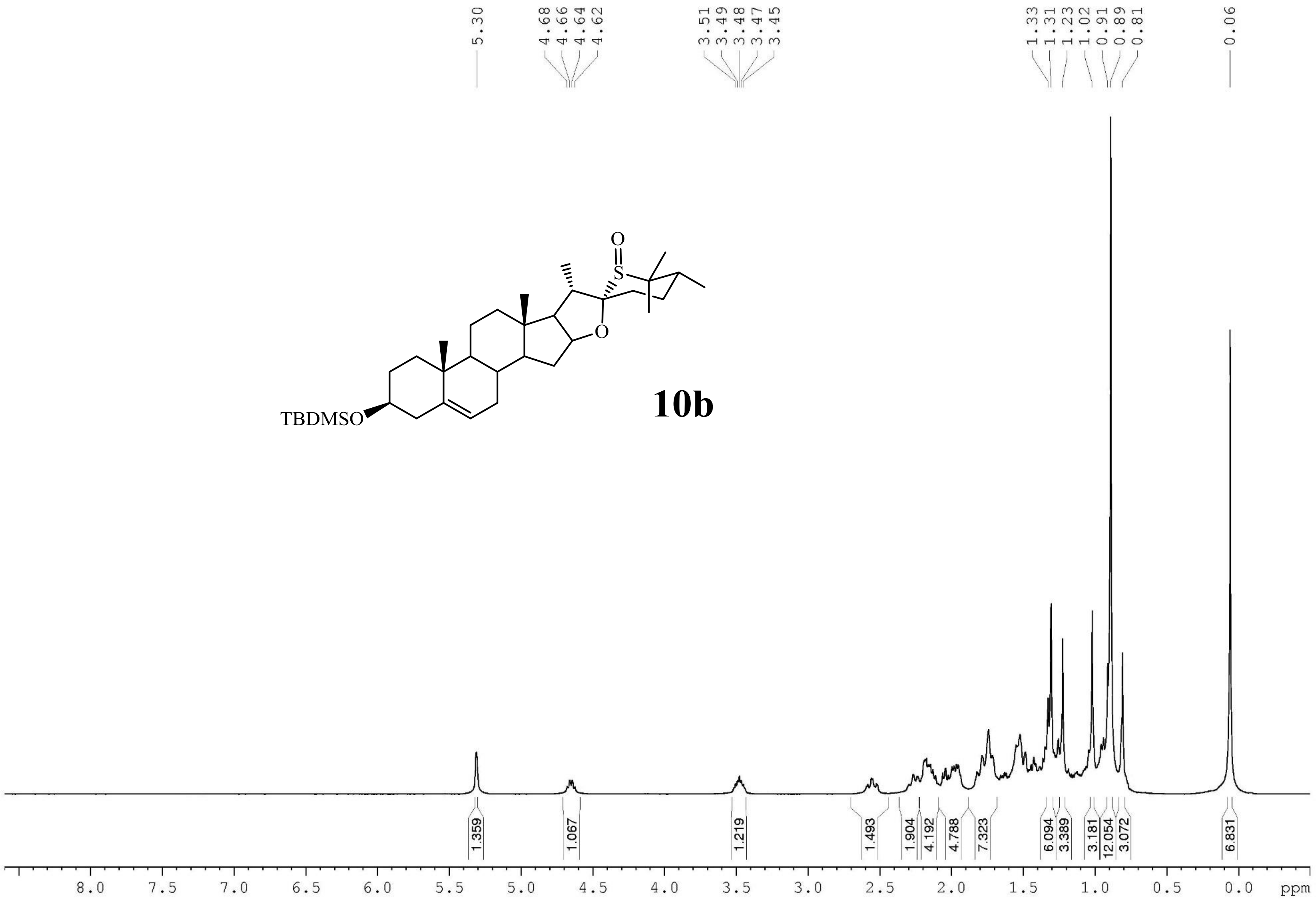
10b
TBDMSO
5.30
4.68
4.66
4.64
4.62
3.51
3.49
3.48
3.47
3.45
1.33
1.31
1.23
1.02
0.91
0.89
0.81
0.06
1.359
1.067
1.219
1.493
1.904
4.192
4.788
7.323
6.094
3.389
3.181
12.054
3.072
6.831
8.0 7.5 7.0 6.5 6.0 5.5 5.0 4.5 4.0 3.5 3.0 2.5 2.0 1.5 1.0 0.5 0.0 ppm

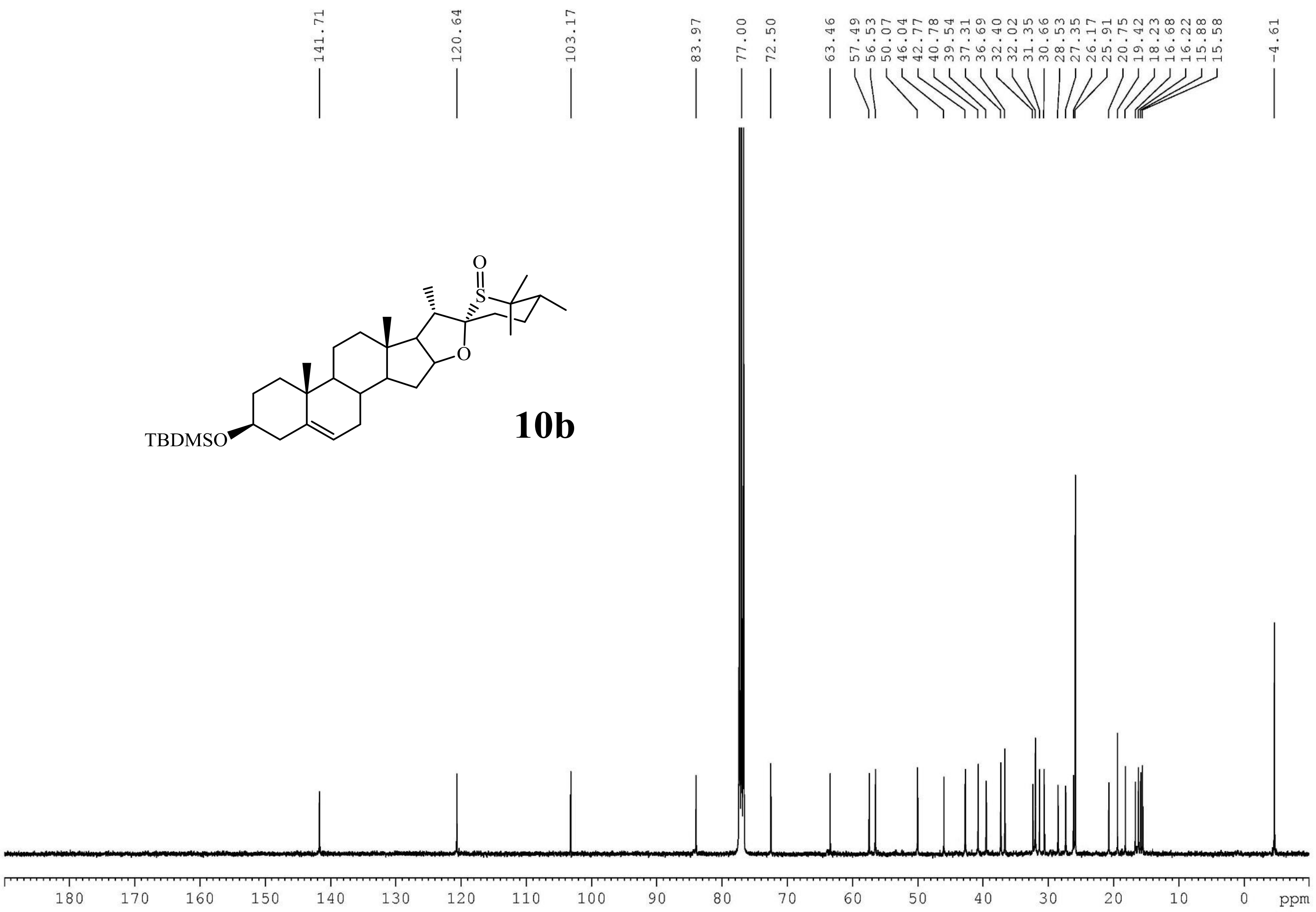
141.71
120.64
103.17
83.97
77.00
72.50
63.46
57.49
56.53
50.07
46.04
42.77
40.78
39.54
37.31
36.69
32.40
32.02
31.35
30.66
28.53
27.35
26.17
25.91
20.75
19.42
18.23
16.68
16.22
15.88
15.58
-4.61
TBDMSO
O
S
O
10b
180 170 160 150 140 130 120 110 100 90 80 70 60 50 40 30 20 10 0 ppm

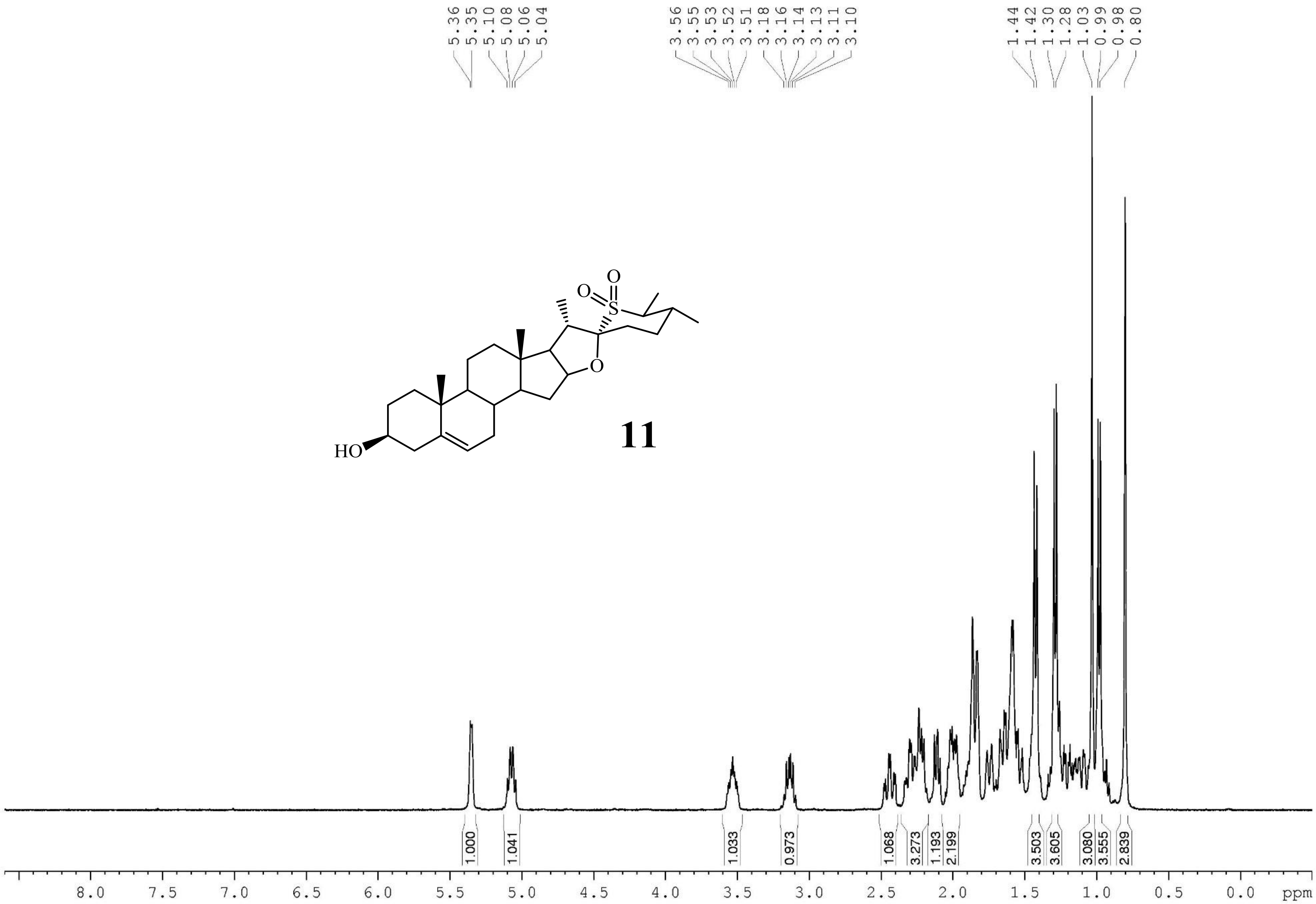
5.36
5.35
5.10
5.08
5.06
5.04
3.56
3.55
3.53
3.52
3.51
3.18
3.16
3.14
3.13
3.11
3.10
1.44
1.42
1.30
1.28
1.03
0.99
0.98
0.80
HO
O
S
O
O
1.000
1.041
1.033
0.973
1.068
3.273
1.193
2.199
3.503
3.605
3.080
3.555
2.839
8.0
7.5
7.0
6.5
6.0
5.5
5.0
4.5
4.0
3.5
3.0
2.5
2.0
1.5
1.0
0.5
0.0
ppm

11

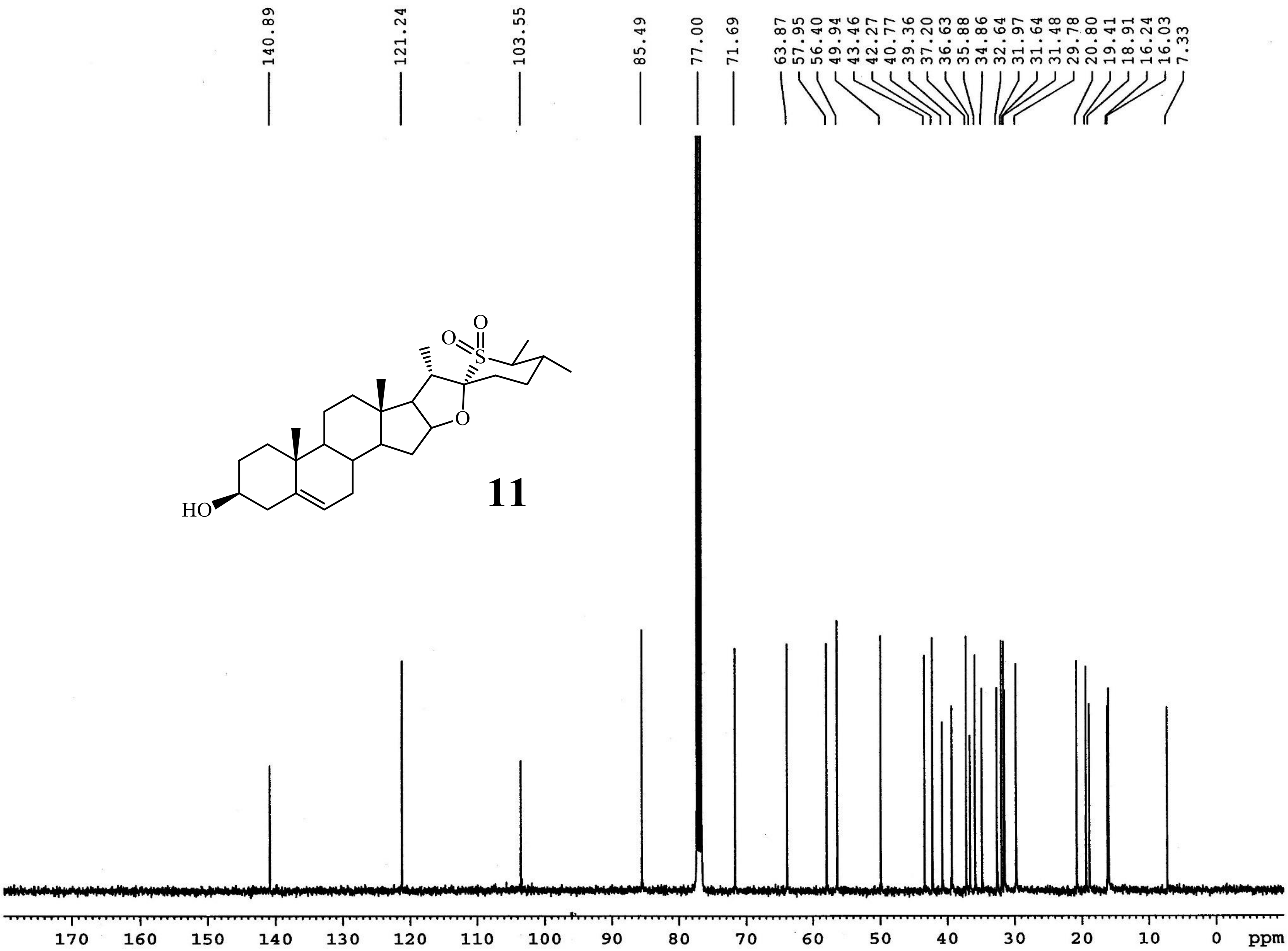
140.89
121.24
103.55
85.49
77.00
71.69
63.87
57.95
56.40
49.94
43.46
42.27
40.77
39.36
37.20
36.63
35.88
34.86
32.64
31.97
31.64
31.48
29.78
20.80
19.41
18.91
16.24
16.03
7.33
HO
O
O
S
O
11
170
160
150
140
130
120
110
100
90
80
70
60
50
40
30
20
10
0
ppm

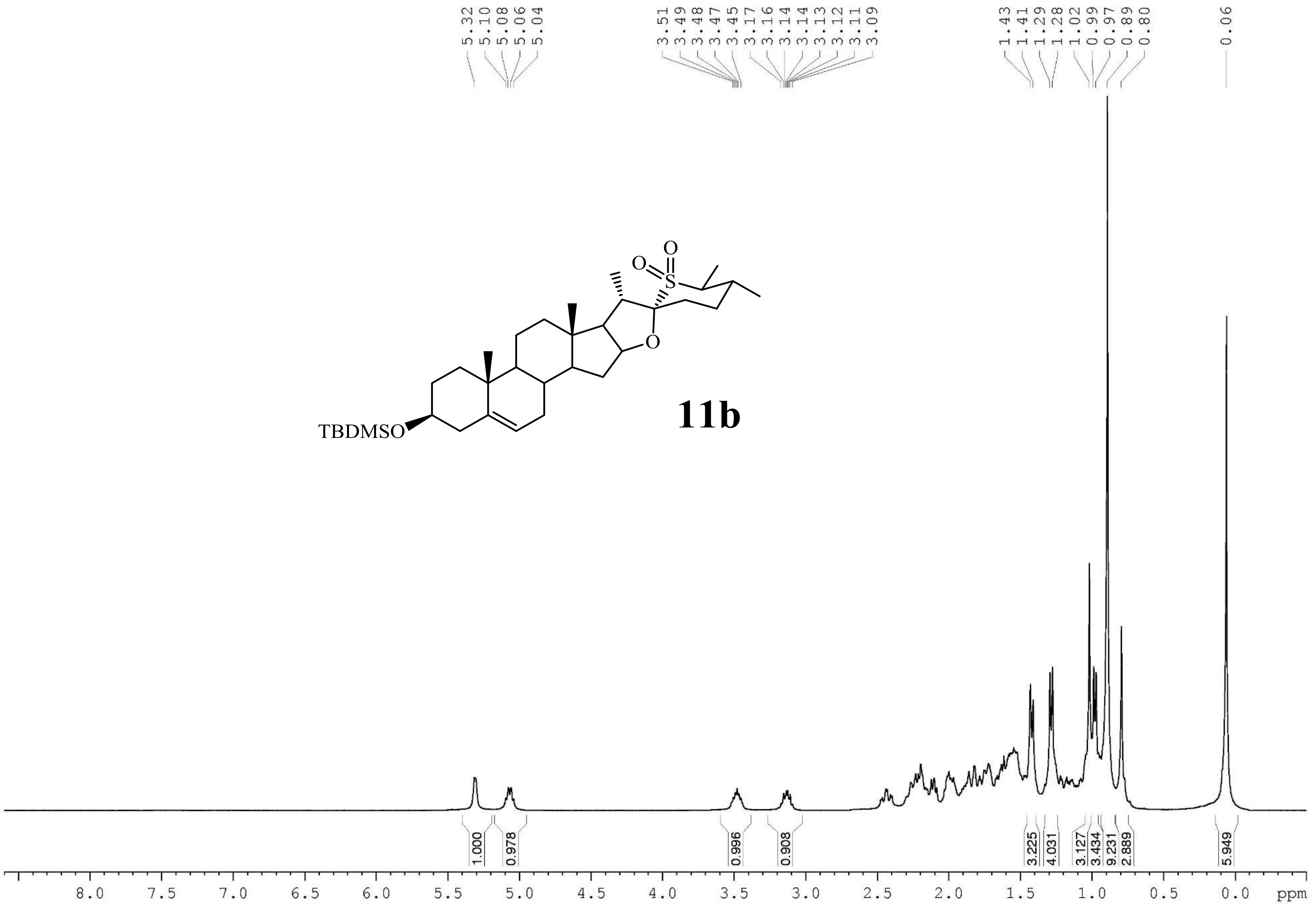
11b

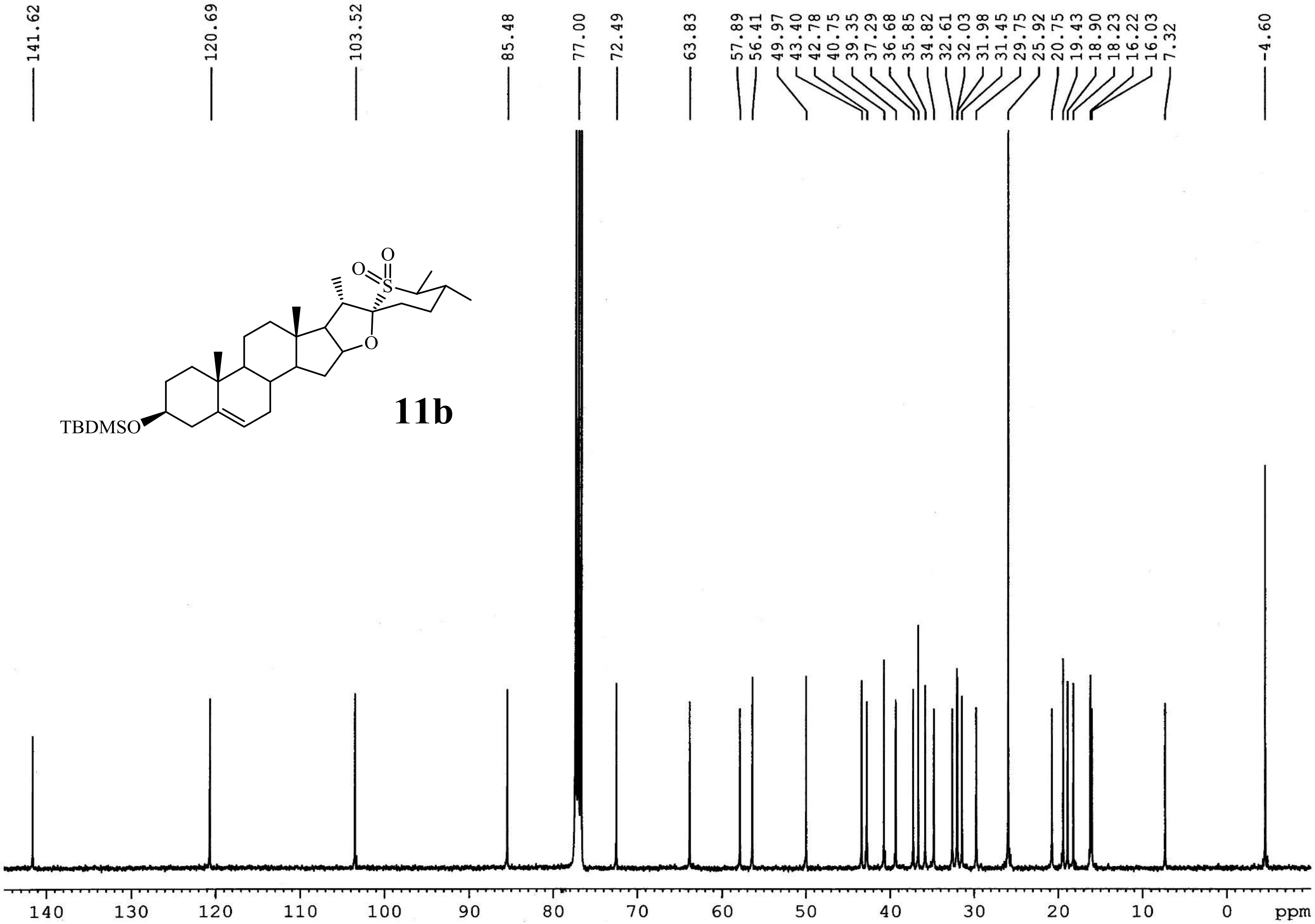
141.62
120.69
103.52
85.48
77.00
72.49
63.83
57.89
56.41
49.97
43.40
42.78
40.75
39.35
37.29
36.68
35.85
34.82
32.61
32.03
31.98
31.45
29.75
25.92
20.75
19.43
18.90
18.23
16.22
16.03
7.32
-4.60
TBDMSO
O
O
S
O
11b
140
130
120
110
100
90
80
70
60
50
40
30
20
10
0
ppm

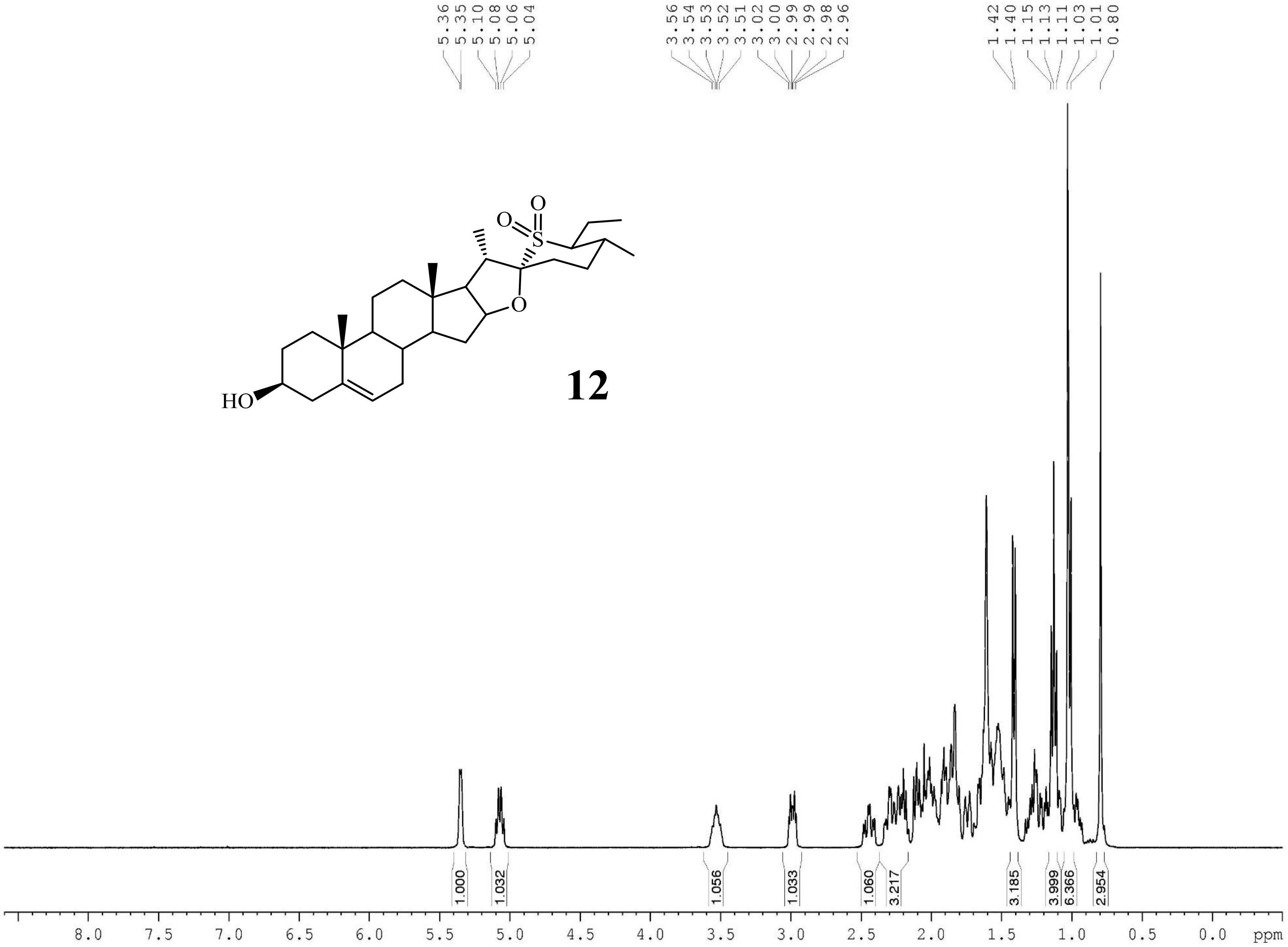
5.36
5.35
5.10
5.08
5.06
5.04
3.56
3.54
3.53
3.52
3.51
3.02
3.00
2.99
2.99
2.98
2.96
1.42
1.40
1.15
1.13
1.11
1.03
1.01
0.80
O
O
S
O
HO
12
1.000
1.032
1.056
1.033
1.060
3.217
3.185
3.999
6.366
2.954
8.0
7.5
7.0
6.5
6.0
5.5
5.0
4.5
4.0
3.5
3.0
2.5
2.0
1.5
1.0
0.5
0.0
ppm

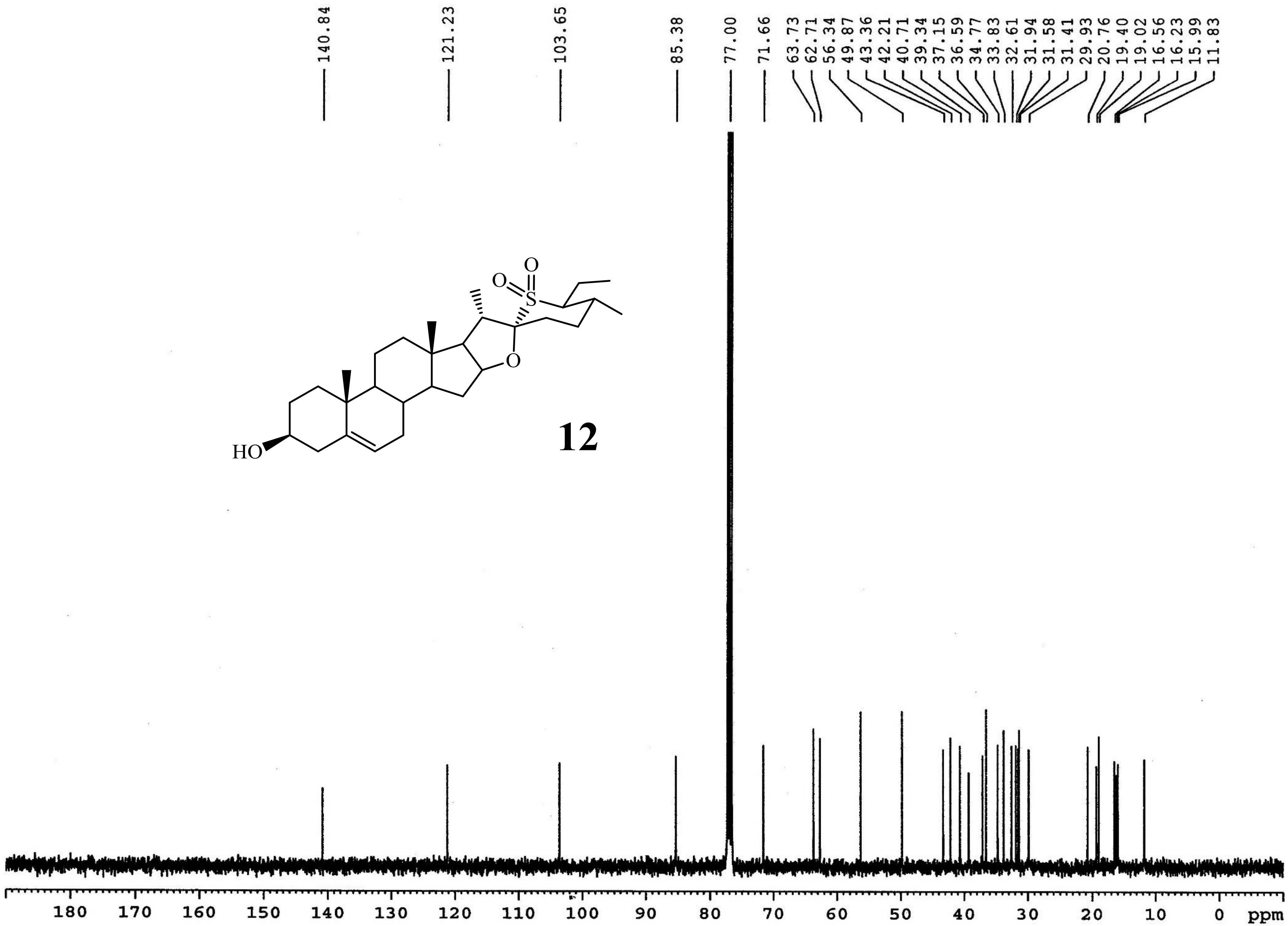
140.84
121.23
103.65
85.38
77.00
71.66
63.73
62.71
56.34
49.87
43.36
42.21
40.71
39.34
37.15
36.59
34.77
33.83
32.61
31.94
31.58
31.41
29.93
20.76
19.40
19.02
16.56
16.23
15.99
11.83
O
O
S
O
HO
12
180 170 160 150 140 130 120 110 100 90 80 70 60 50 40 30 20 10 0 ppm

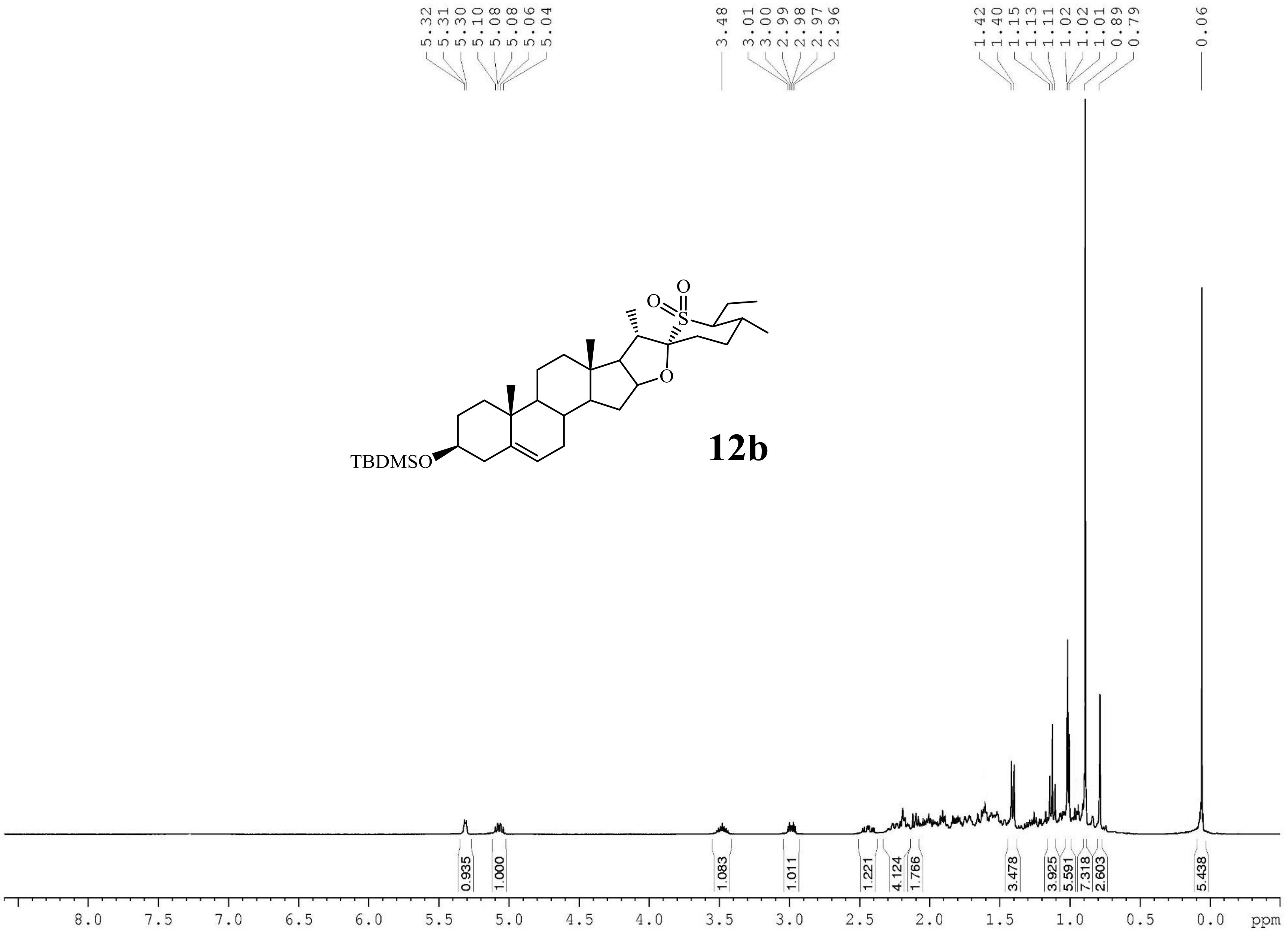
5.32
5.31
5.30
5.10
5.08
5.08
5.06
5.04
3.48
3.01
3.00
2.99
2.98
2.97
2.96
1.42
1.40
1.15
1.13
1.11
1.02
1.02
1.01
0.89
0.79
0.06
TBDMSO
O
O
S
O
12b
0.935
1.000
1.083
1.011
1.221
4.124
1.766
3.478
3.925
5.591
7.318
2.603
5.438
8.0 7.5 7.0 6.5 6.0 5.5 5.0 4.5 4.0 3.5 3.0 2.5 2.0 1.5 1.0 0.5 0.0 ppm

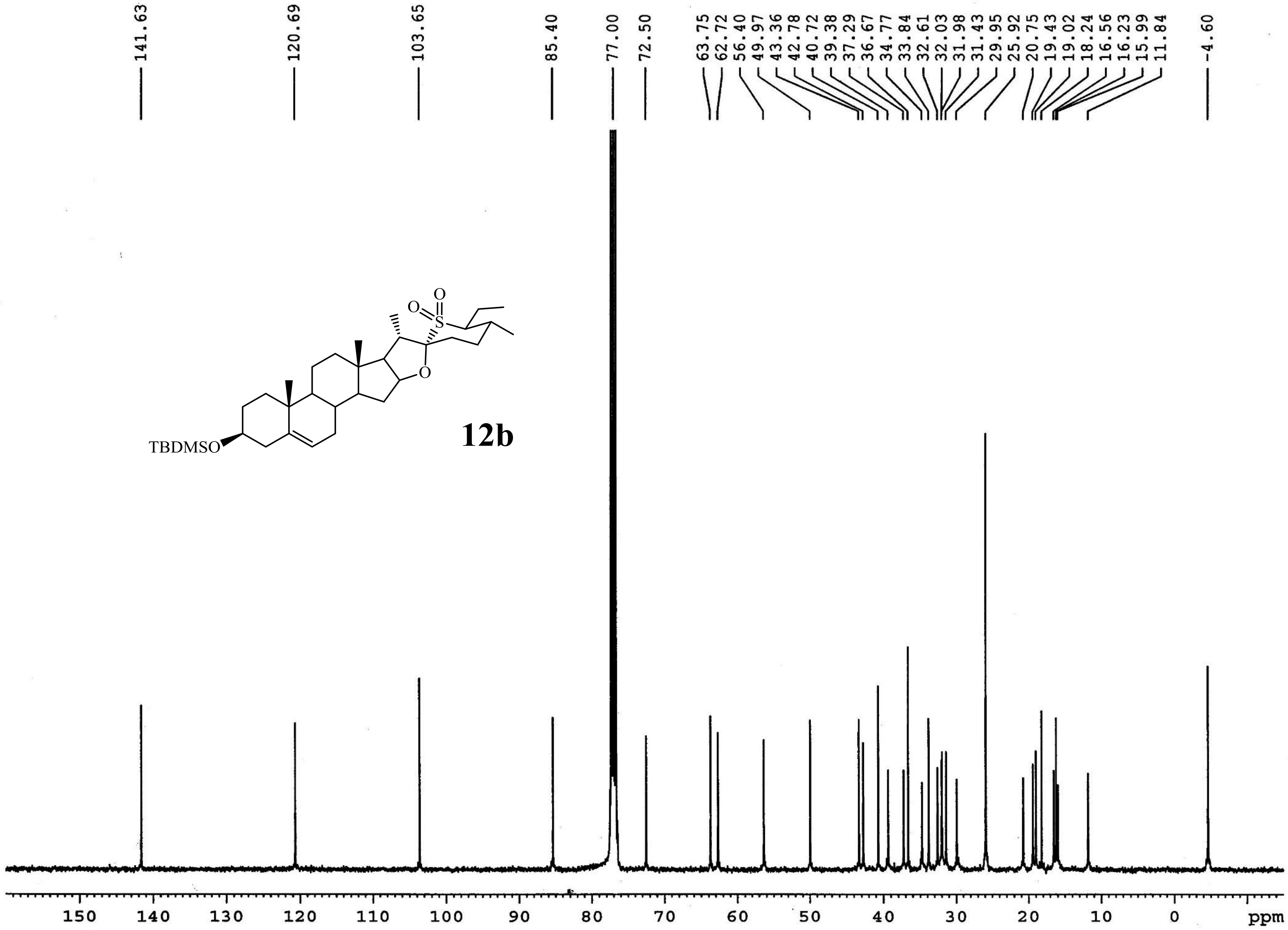
141.63
120.69
103.65
85.40
77.00
72.50
63.75
62.72
56.40
49.97
43.36
42.78
40.72
39.38
37.29
36.67
34.77
33.84
32.61
32.03
31.98
31.43
29.95
25.92
20.75
19.43
19.02
18.24
16.56
16.23
15.99
11.84
-4.60
TBDMSO
O
S
O
O
12b
150
140
130
120
110
100
90
80
70
60
50
40
30
20
10
0
ppm
